# High-Throughput Parallel Sequencing to Measure Fitness of *Leptospira interrogans* Transposon Insertion Mutants during Acute Infection

**DOI:** 10.1371/journal.pntd.0005117

**Published:** 2016-11-08

**Authors:** Kristel Lourdault, James Matsunaga, David A. Haake

**Affiliations:** 1 Veterans Affairs Greater Los Angeles Healthcare System, Los Angeles, California, United States of America; 2 Departments of Medicine, David Geffen School of Medicine at University of California Los Angeles, Los Angeles, California, United States of America; 3 Departments of Urology, David Geffen School of Medicine at University of California Los Angeles, Los Angeles, California, United States of America; 4 Departments of Microbiology, Immunology, and Molecular Genetics, University of California Los Angeles, Los Angeles, California, United States of America; Mahidol University, THAILAND

## Abstract

Pathogenic species of *Leptospira* are the causative agents of leptospirosis, a zoonotic disease that causes mortality and morbidity worldwide. The understanding of the virulence mechanisms of *Leptospira spp* is still at an early stage due to the limited number of genetic tools available for this microorganism. The development of random transposon mutagenesis in pathogenic strains a decade ago has contributed to the identification of several virulence factors. In this study, we used the transposon sequencing (Tn-Seq) technique, which combines transposon mutagenesis with massive parallel sequencing, to study the *in vivo* fitness of a pool of *Leptospira interrogans* mutants. We infected hamsters with a pool of 42 mutants (input pool), which included control mutants with insertions in four genes previously analyzed by virulence testing (*loa22*, *ligB*, *flaA1*, and *lic20111*) and 23 mutants with disrupted signal transduction genes. We quantified the mutants in different tissues (blood, kidney and liver) at 4 days post-challenge by high-throughput sequencing and compared the frequencies of mutants recovered from tissues to their frequencies in the input pool. Control mutants that were less fit in the Tn-Seq experiment were attenuated for virulence when tested separately in the hamster model of lethal leptospirosis. Control mutants with unaltered fitness were as virulent as the wild-type strain. We identified two mutants with the transposon inserted in the same putative adenylate/guanylate cyclase gene (*lic12327*) that had reduced *in vivo* fitness in blood, kidney and liver. Both *lic12327* mutants were attenuated for virulence when tested individually in hamsters. Growth of the control mutants and *lic12327* mutants in culture medium were similar to that of the wild-type strain. These results demonstrate the feasibility of screening large pools of *L*. *interrogans* transposon mutants for those with altered fitness, and potentially attenuated virulence, by transposon sequencing.

## Author Summary

Leptospirosis is a neglected infectious disease that sickens many humans and animals throughout the world. It is caused by pathogenic *Leptospira spp*. Few leptospiral genes that contribute to the disease have been identified. We generated a library of 800 *L*. *interrogans* mutants with transposon insertions in different genes. Screening each mutant individually for the ability to cause disease in the hamster model would be laborious and requires thousands of animals. In a pilot experiment, we infected hamsters with a pool of 42 mutants to determine the role of the disrupted genes on fitness of the bacterium during infection. Out of the 12 mutants with diminished fitness in the three tissues, two had transposon insertions in the gene encoding an enzyme that may generate the small signaling molecule cAMP. When tested separately, the two mutants failed to sicken hamsters, indicating that intracellular cAMP signaling within *L*. *interrogans* could have a role in causing disease. These findings indicate that large pools of transposon insertion mutants can be screened in a limited number of animals to identify leptospiral genes that may be critical for the disease process.

## Introduction

Pathogenic *Leptospira spp* are the causative agents of leptospirosis, presumed to be the most widespread zoonotic disease [1]. Leptospirosis has emerged as a major public health burden in urban slums where risk is strongly linked to poverty and rat exposure [2–4]. It is estimated that there are 1.03 million cases and 58,900 deaths each year from leptospirosis [5]. The disease is transmitted to humans and animals through the urine of infected animals such as rats [1, 6]. Bacteria enter the host via skin abrasions or mucous membranes and then disseminate via the bloodstream to target organs including the lungs, liver and kidneys. Infection produces a range of clinical manifestations, from flu-like symptoms to liver dysfunction, bleeding, kidney failure, pulmonary hemorrhage, and occasionally death [6, 7].

The understanding of the virulence mechanisms of *Leptospira spp* is still at an early stage compared to other bacteria due to the limited number of genetic tools available for leptospires. The sequencing of a large number of leptospiral genomes [8] reveals that genes encoding proteins of unknown functions are enriched among pathogen-specific leptospiral genes [9]. Development of random transposon mutagenesis in pathogenic strains a decade ago has contributed to a better understanding of *Leptospira* biology [10] and has enabled identification of several virulence genes, including *loa22*, the first leptospiral virulence gene to be described [11], and *lb139*, a gene encoding a potential signaling protein [12]. Interestingly, attenuation of virulence did not occur following inactivation of *ligB* or other genes whose products have been shown to have virulence attributes *in vitro* [13]. These results suggest a large degree of functional redundancy of virulence-associated gene products [6, 14].

A disadvantage of virulence testing of individual transposon mutants is that this approach requires the use of a large number of animals. Although animal models remain critical for understanding leptospiral pathogenesis and for identifying virulence factors, there is a need for new approaches to reduce the number of animals required for such experiments.

One strategy to minimize the number of animals is to inoculate pools of mutants into each animal. Recently, Marcsisin *et al*. screened pools of defined *L*. *interrogans* transposon insertion mutants for infectivity in the hamster model of acute infection [15]. 95 mutants were tested in pools of up to 10 mutants. 25 mutants were also tested in pools of 5 mutants in the mouse carrier model of infection. This study focused on whether mutants could be detected by PCR in cultures obtained from blood and kidney. Mutants with severe infectivity defects were tested individually for lethal virulence in hamsters. Only one mutant that failed to cause mortality was identified, although death was delayed with another four mutants. Because the level of each mutant was not quantified in the tissues, the approach was biased towards identification of highly attenuated mutants [15].

Transposon sequencing (Tn-Seq) has the potential to identify virulence-attenuated mutants with more subtle effects on infectivity. This technique combines transposon mutagenesis with the power of massive parallel sequencing. The basic principle of transposon sequencing methods involves DNA extraction from the pool of mutants, its cleavage by restriction enzyme digestion or sonication and the addition of adaptors for PCR amplification of the transposon ends and flanking regions. The PCR amplicons are analyzed by high throughput sequencing to determine the insertion site of each mutant and their relative abundance [16–18]. Tn-Seq has been used to study *in vivo* fitness of various bacteria such as *Burkholderia pseudomallei* [19], *Streptococcus pneumoniae* [16], *Haemophilus influenza* [20], and *Borrelia burgdorferi* [21, 22] and to identify bottlenecks during mouse infection by *B*. *burgdorferi* [23]. Tn-Seq has also been used to identify genes contributing to *in vitro* phenotypes, including antibiotic resistance in *Pseudomonas aeruginosa* [22] and carbon utilization in *B*. *burgdorferi* [21].

In the present study, we examined the potential of Tn-Seq to quantify the fitness of a pool of leptospiral mutants in various tissues during acute infection of the hamster. We screened mutants with transposon insertions in signal transduction genes to determine whether these genes affect the fitness of *L*. *interrogans*. The virulence of selected mutants with reduced fitness was tested in the hamster model of acute leptospirosis.

## Materials and Method

### Bacterial strains and growth medium

The pathogen *Leptospira interrogans* serovar Manilae strain L495 was used as the parent strain for generation of a transposon mutant library. The wild type (WT) strain and all mutants (Table 1) derived from it were grown at 30°C in Ellinghausen-McCullough-Johnson-Harris (EMJH) medium [24, 25] and EMJH supplemented with kanamycin (Km, 50 mg/mL), respectively.

**Table 1:** Transposon mutants used in this study.

LIC number	Chr*	Insertion sites**	Name and Description	
**Non-signaling genes**				
lic10191	chrI	220833	*loa22*—control	OmpA-family lipoprotein
lic10203	chrI	232860	sugar epimerase	Nucleoside-diphosphate-sugar epimerase
lic10464	chrI	529904	*ligB*—control	Bacterial immunogloblin
lic10788	chrI	955881	*flaA1*—control	Endoflagellar filament sheath protein
lic11081	chrI	1336016	*lolD*	Lipoprotein releasing system, LolD ATPase component
lic11274	chrI	1574819	hypothetical protein	hypothetical protein
lic11889	chrI	2286578	*flaB2*	Endoflagellar filament core protein
lic11940	chrI	2349558	AcrA efflux pump	AcrA-related membrane protein
lic12772	chrI	3379463	proB glutamate kinase	Glutamate 5-kinase
lic12773	chrI	3379503	GTPase	GTPase
lic13074	chrI	3756887	AcrB efflux pump	Efflux pump, AcrB family
lic13274	chrI	4015561	hypothetical protein	hypothetical protein
lic20148	chrII	179873	heme oxygenase	Heme oxygenase
**Signal transduction genes**				
**Adenylate/guanylate cyclases**				**Domains**
lic10024	chrI	30718	adenylate/guanylate cyclase	7TMR, AC/GC
lic11095	chrI	1352244	adenylate/guanylate cyclase	AC/GC
lic12327a	chrI	2810027	adenylate/guanylate cyclase	GAF, AC/GC
lic12327b	chrI	2810271	adenylate/guanylate cyclase	GAF, AC/GC
lic12506	chrI	3035366	adenylate/guanylate cyclase	FeS, AC/GC, Heme peroxidase
lic12670	chrI	3232667	adenylate/guanylate cyclase	HAMP, AC/GC
lic13004	chrI	3653040	adenylate/guanylate cyclase	AC/GC
**Diguanylate cyclases/phosphodiesterases**				
lic10138	chrI	164403	HD-GYP hydrolase domain-containing protein	HY-GYP
lic10641	chrI	783180	Signal transduction protein	EAL
lic20182	chrII	230752	GGDEF family protein	Cache_1, GGDEF
**Histidine kinase**				
lic11432	chrI	1759905	Sensor histidine kinase and response regulator of a two component complex	PAS, PAS, HK, REC
lic12031	chrI	2450863	Sensor protein of a two component response regulator	PAS, PAS, PAS, PAS, HK
lic12218	chrI	2676839	Sensor histidine kinase of a two component response	HK
lic12627a	chrI	3177595	Histidine kinase of a two-component regulator system	REC, PAS, HK
lic12627b	chrI	3178505	Histidine kinase of a two-component regulator system	REC, PAS, HK
**Transcriptional regulator**				
lic13073	chrI	3754434	Transcriptional regulator, AcrR-family	HTH (TetR family)
**Alternative σ factor**				
lic10132	chrI	158862	Transcriptional regulator	GAF, σ54 activator, HTH_8
lic10225	chrI	258550	RNA polymerase sigma subunit	ECF-type σ factor
lic12502	chrI	3030630	Sigma 54 modulation protein / S30EA ribosomal protein	σ54 modulation protein
**Phosphatase**				
lic12324a	chrI	2806250	Signal transduction protein with multiple domains	PK, AAA_16, GAF, SpoIIE
lic12324b	chrI	2806718	Signal transduction protein with multiple domains	PK, AAA_16, GAF, SpoIIE
lic20111	chrII	132731	Regulator of sigma subunit—control	HAMP, SpoIIE
**Other**				
lic11563	chrI	1923334	Response regulator with HD-GYP domain	DUF3391, HD (phosphohydrolase)
**Intergenic regions**				
inter10855	chrI	953546	Intergenic—between lepic0802/lic10786 genes	
inter11063	chrI	1175475	Intergenic—between lic10973/lic10974 genes	
inter12760	chrI	3104330	Intergenic—between lic12561/lepic2609 genes	
inter13512	chrI	4004549	Intergenic—between lic13264/lepi3326 genes	
inter13722	chrI	4230621	Intergenic—between lic13452/lic13453 genes	
Inter20138	chrII	159644	Intergenic—between lepic0137/lic20136 genes	

*chr: chromosome (I or II)

** nucleotide of the insertion site in the Fiocruz L1-130 genome.

*Escherichia coli* strain β2163 [26] containing the shuttle vector (pCjTKS2) [27], which carries a *Himar1* transposon, was grown at 37°C in Luria broth supplemented with 2,3-diaminopimelic acid (DAP, 0.3 mM), kanamycin (Km, 50 mg/mL) and spectinomycin (Spc, 50 mg/mL).

### Creation of a leptospiral mutant library, and identification of insertion sites

An L495 mutant library was generated by random transposon insertion mutagenesis [28]. Briefly, the shuttle vector pCjTKS2, which contains a *Himar1* element with its transposase gene lying outside of the transposon, was introduced into the L495 strain by conjugation with the *E*. *coli* β2163 donor strain. After two to three weeks of growth at 30°C on EMJH+Km plates, colonies were inoculated into EMJH+Km liquid medium and grown at 30°C for three to four weeks. Mutants were separately frozen at -80°C in EMJH and 4% glycerol (final concentration) without passaging.

For each mutant, the insertion site of the transposon in the genome was determined by semi-random PCR as previously described by Slamti *et al*. [28]. PCR primer sequences are provided in the Table 2. The insertion sites were identified by comparing the resulting sequence with the *L*. *interrogans* serovar Copenhageni Fiocruz L1-130 genome using the SpiroScope database (http://www.genoscope.cns.fr/agc/mage) [29].

**Table 2:** Primers used in this study.

Name	Sequence (5'-3')	Reference
**Primers used for semi-random PCR**		
**TnK1**	CTTGTCATCGTCATCCTTG	[28]
**Tnk2**	GTGGCTTTATTGATCTTGGG	
**Deg1**	GGCCACGCGTCGACTAGTAC**NNNNNNNNNN**GATAT	
**Deg2**	GGCCACGCGTCGACTAGTAC**NNNNNNNNNN**TCTT	
**TnkN1**	CGTCATGGTCTTTGTAGTCTATGG	
**TnKN2**	TGGGGATCAAGCCTGATTGGG	
**Tag**	GGCCACGCGTCGACTAGTAC	
**Primers used for Illumina sequencing**		
**TnkN3**	CGGGGAAGAACAGTATGTCGAGCTATTTTTTGACTTACTGGGGATCAAGCCTGATTGGG	This study
**olj376**	GTGACTGGAGTTCAGACGTGTGCTCTTCCGATCTGGGGGGGGGGGGGGGG	[23]
**pMargent2**	AATGATACGGCGACCACCGAGATCTACACTCTTTCCGGGGACTTATCAGCCAACCTGTTA	
**IP 1**	CAAGCAGAAGACGGCATACGAGAT**CGTGAT**GTGACTGGAGTTCAGACGTGTGCTCTTCCGATCT	Illumina sequencing (barcodes in bold)
**IP 2**	CAAGCAGAAGACGGCATACGAGAT**ACATCG**GTGACTGGAGTTCAGACGTGTGCTCTTCCGATCT	
**IP 3**	CAAGCAGAAGACGGCATACGAGAT**GCCTAA**GTGACTGGAGTTCAGACGTGTGCTCTTCCGATCT	
**IP 4**	CAAGCAGAAGACGGCATACGAGAT**TGGTCA**GTGACTGGAGTTCAGACGTGTGCTCTTCCGATCT	
**IP 5**	CAAGCAGAAGACGGCATACGAGAT**CACTGT**GTGACTGGAGTTCAGACGTGTGCTCTTCCGATCT	
**IP 6**	CAAGCAGAAGACGGCATACGAGAT**ATTGGC**GTGACTGGAGTTCAGACGTGTGCTCTTCCGATCT	
**IP 7**	CAAGCAGAAGACGGCATACGAGAT**GATCTG**GTGACTGGAGTTCAGACGTGTGCTCTTCCGATCT	
**IP 8**	CAAGCAGAAGACGGCATACGAGAT**TCAAGT**GTGACTGGAGTTCAGACGTGTGCTCTTCCGATCT	
**IP 9**	CAAGCAGAAGACGGCATACGAGAT**CTGATC**GTGACTGGAGTTCAGACGTGTGCTCTTCCGATCT	
**IP 10**	CAAGCAGAAGACGGCATACGAGAT**AAGCTA**GTGACTGGAGTTCAGACGTGTGCTCTTCCGATCT	
**IP 11**	CAAGCAGAAGACGGCATACGAGAT**GTAGCC**GTGACTGGAGTTCAGACGTGTGCTCTTCCGATCT	
**IP 12**	CAAGCAGAAGACGGCATACGAGAT**TACAAG**GTGACTGGAGTTCAGACGTGTGCTCTTCCGATCT	
**IP 13**	CAAGCAGAAGACGGCATACGAGAT**TTGACT**GTGACTGGAGTTCAGACGTGTGCTCTTCCGATCT	
**IP 14**	CAAGCAGAAGACGGCATACGAGAT**GGAACT**GTGACTGGAGTTCAGACGTGTGCTCTTCCGATCT	
**IP 15**	CAAGCAGAAGACGGCATACGAGAT**TGACAT**GTGACTGGAGTTCAGACGTGTGCTCTTCCGATCT	
**IP 16**	CAAGCAGAAGACGGCATACGAGAT**GGACGG**GTGACTGGAGTTCAGACGTGTGCTCTTCCGATCT	
**IP 17**	CAAGCAGAAGACGGCATACGAGAT**CTCTAC**GTGACTGGAGTTCAGACGTGTGCTCTTCCGATCT	
**IP 18**	CAAGCAGAAGACGGCATACGAGAT**GCGGAC**GTGACTGGAGTTCAGACGTGTGCTCTTCCGATCT	
**IP 19**	CAAGCAGAAGACGGCATACGAGAT**TTTCAC**GTGACTGGAGTTCAGACGTGTGCTCTTCCGATCT	
**IP 20**	CAAGCAGAAGACGGCATACGAGAT**GGCCAC**GTGACTGGAGTTCAGACGTGTGCTCTTCCGATCT	
**IP 21**	CAAGCAGAAGACGGCATACGAGAT**CGAAAC**GTGACTGGAGTTCAGACGTGTGCTCTTCCGATCT	
**IP 22**	CAAGCAGAAGACGGCATACGAGAT**CGTACG**GTGACTGGAGTTCAGACGTGTGCTCTTCCGATCT	
**IP 23**	CAAGCAGAAGACGGCATACGAGAT**CCACTC**GTGACTGGAGTTCAGACGTGTGCTCTTCCGATCT	
**IP 24**	CAAGCAGAAGACGGCATACGAGAT**GCTACC**GTGACTGGAGTTCAGACGTGTGCTCTTCCGATCT	
**IP 25**	CAAGCAGAAGACGGCATACGAGAT**ATCAGT**GTGACTGGAGTTCAGACGTGTGCTCTTCCGATCT	
**IP 26**	CAAGCAGAAGACGGCATACGAGAT**GCTCAT**GTGACTGGAGTTCAGACGTGTGCTCTTCCGATCT	
**IP 27**	CAAGCAGAAGACGGCATACGAGAT**AGGAAT**GTGACTGGAGTTCAGACGTGTGCTCTTCCGATCT	
**IP 28**	CAAGCAGAAGACGGCATACGAGAT**CTTTTG**GTGACTGGAGTTCAGACGTGTGCTCTTCCGATCT	
**IP 29**	CAAGCAGAAGACGGCATACGAGAT**TAGTTG**GTGACTGGAGTTCAGACGTGTGCTCTTCCGATCT	
**IP seq**	GATCGGAAGAGCACACGTCTGAACTCCAGTCAC	
**pMargent3**	ACACTCTTTCCGGGGACTTATCAGCCAACCTGTTA	[23]
**Primers used for quantitative PCR**		
**LipL32-45F**	AAGCATTACCGCTTGTGGTG	[30]
**LipL32-286R**	GAACTCCCATTTCAGCGATT	
**LipL32-189P**	[6-FAM]AAAGCCAGGACAAGCGCCG[BHQ1a-Q]	
**Primers used for quantitative Real time PCR**		
**RTlic20110-F**	ATCCGCCTTTTAGAACACGC	This study
**RTlic20110-R**	TTTGCAAATACGGTTCCGGG	
**RTlic20109-F**	AACCGGTATCTCAGAGCCTC	
**RTlic20109-R**	GAACCGAAATGCCACCTTCA	
**RTlic20108-F**	ATTGATGAAGGGGCGAGAGT	
**RTlic20108-R**	AATCGGGCTAGGAATTGCGT	
**RTrpoB-F**	ATGGAGCGGAACGTGTAGTC	[31]
**RTrpoB-R**	CTTCGTTCGTTCCATGTCCT	
**RTflaB-F**	GAGAGAAACACCGAAGACGG	[32]
**RTflaB-R**	TGAATAGCAAGAACCCGGAT	

### Ethics statement and endpoint criteria

All animals were routinely cared for according to the guidelines provided in the National Institutes of Health Guide to Laboratory Animal Care. Procedures involving hamsters were approved by the Veterans Affairs Greater Los Angeles Healthcare System Institutional Animal Care and Use Committee (protocol #09018–14). Hamsters were weighed daily and observed for endpoint criteria, including loss of appetite, gait or breathing difficulty, prostration, ruffled fur, or weight loss of >10% of maximum weight. Animals that met any of the endpoint criteria were euthanized by isoflurane inhalation followed by thoracotomy.

### Animal experiments

#### Tn-Seq

For this experiment, 42 L495 mutants with *Himar1* insertions in 33 ORFs and 6 intergenic regions were selected (Table 1) and recovered from storage at -80°C by growing them individually in EMJH+Km at 30°C. On the challenge day, leptospires were counted by dark-field microscopy as described by Miller [33]. Briefly 10 μl of each culture was diluted into 490 μl PBS. 10 μl of the suspension was placed on a slide and covered with a coverslip, and the bacterial cells counted with the 40x objective under an Axio Lab A1 microscope with a darkfield condenser (Zeiss). Bacterial counts from five fields were averaged, and the culture density was calculated based on the diameter of the field of view and the volume of suspension under the coverslip. Cultures were then diluted in EMJH to a concentration of 10^6^/mL and pooled together in equal amounts.

Eight four-week-old female Golden Syrian hamsters (Envigo RMS) were challenged intraperitoneally with 1 mL of the pool of mutants, i.e 10^6^ leptospires total. Hamsters were monitored daily until day 4 when they were terminated. At the time of sacrifice, kidney and liver were collected into cryotubes and stored at -80°C until use. Blood was collected in EDTA (BD vacutainer Plus Plastic K_2_ EDTA tubes), and DNA was immediately extracted (see below). A fraction of the blood was collected in dry tubes and the serum was obtained by centrifugation at 21,130 x g for 15 min and stored at -80°C until use.

On the challenge day, 10 mL of the input pool was spun down for 20 min at 3,220 x g, and the pellet was stored at -80°C until use.

#### Survival experiment

Hamsters in groups of six were challenged intraperitoneally with 10^6^ leptospires: *loa22*::Tn, *ligB*::Tn, *flaA1*::Tn, *lic20111*::Tn, *lic12327a*::Tn, *lic12327b*::Tn or the WT strain. The animals were monitored daily until endpoint criteria were met or for 28 days, at which time they were euthanized. At the time of sacrifice, one kidney was collected and cultured in semisolid Probumin Vaccine-grade Solution (Millipore) containing 100 μg/mL of 5-fluorouracil and 0.2% Bacto-Agar at 30°C. Cultures were checked weekly for a month for leptospiral growth.

### Construction of genomic library for sequencing

Genomic libraries for sequencing were constructed as described by Troy *et al*. [23]. Genomic DNA was extracted from 100 μl of blood, 25 mg of tissue or frozen pellet from input pool with the DNeasy blood and tissue kit (Qiagen, Valencia, CA) following the manufacturer’s instructions except that an elution volume of only 100 μl was used. Extracted DNA was stored at -80°C until use.

50 μl of extracted DNA was sheared by sonication with a Fisher Scientific Model 505 Sonic Dismembrator for 3 min (10 s on and 5 s off; intensity, 80%) in a high-intensity cup horn that was cooled at 4°C. Cytosine tails (C-tails) were added to 500 ng of sheared DNA using terminal deoxynucleotidyl transferase (TdT) (Promega, Madison, WI). The TdT reaction mixture containing 475 μM dCTP and 25 μM ddCTP (Affymetrix/USB Products, Santa Clara, CA) was incubated for 1 h at 37°C followed by 20 min at 75°C. The DNA was then purified using the Qiagen MinElute PCR Purification kit (Qiagen, Valencia, CA) following the manufacturer’s instructions.

The insertion site of the transposon was amplified by nested PCR. The first PCR was performed with 3 μl of the C-tailed DNA as template using olj376 and TnKN3 primers (Table 2) specific for the C-tail and the *Himar1* transposon, respectively, in a final volume of 25 μl. Primer olj376, at the concentration of 1.8 μM, was added at three times in excess of TnKN3 (600 nM). Reactions were performed using DreamTaq Master Mix (Thermo Scientific) with an initial incubation of 2 min at 95°C followed by 24 cycles of 30 s at 95°C, 30 s at 60°C, and 2 min at 72°C followed by a 2-min extension at 72°C. The second PCR was performed with 2 μl from the previous PCR step with pMArgent2 primer (600 nM) specific for the end of the transposon and an indexing primer (600 nM) containing the specific sequences required for sequencing on an Illumina platform and a six-base-pair barcode sequence allowing all 37 samples to be multiplexed in a single sequencing lane (Table 2). PCR reactions were performed using DreamTaq Master Mix (Thermo Scientific) with an initial incubation of 2 min at 95°C followed by 18 cycles of 30 s at 95°C, 30 s at 60°C, and 2 min at 72°C followed by a 2-min extension at 72°C. PCR products were purified using a QIAquick PCR purification kit (Qiagen, Valencia, CA) following the manufacturer’s instructions except that an elution volume of only 30 μl was used. The majority of PCR products were between 200 bp and 600 bp in size. The DNA concentration of each culture and tissue library was measured with the Qubit 2.0 fluorometer (Thermo Fisher). Equal amounts of DNA from each library were then pooled together and kept at -80°C until sequencing.

### Sequencing and data analysis

The pooled libraries were sequenced on an Illumina HiSeq 2500 next generation sequencing system at the UCLA Neurosciences Genomics core facility as 64 bp single-end reads using the custom sequencing primer pMargent3 and the standard Illumina sequencing primer (Table 2).

Data analysis was performed using the UCLA Galaxy platform [34–37]. Reads were cleaned by removal of ambiguous nucleotides, adapters, and primer sequences. The reads were filtered for length and quality: reads fewer than 20 nucleotides long or with a quality score of 20 or less for 95% of the cycles were eliminated.

The remaining reads were mapped to the *L*. *interrogans* serovar Copenhageni strain Fiocruz L1-130 genome using Bowtie [38]. The resulting file was sorted to obtain a list of insertion sites, their corresponding gene numbers, and the number of reads per insertion site. In this way, the frequency with which each mutant occurred in each tissue and each animal was determined. Output/input ratios for each mutant were calculated by dividing a mutant’s output frequency by its frequency in the input pool.

### Statistics

Output/input ratios across the 42 mutants were normalized by setting the median ratio for each animal to 1.0. Ratios were compared to 1.0 (neutral fitness) using the Wilcoxon rank test with P values < 0.05 considered statistically significant. Comparison of ratios between duplicates was performed using the Student’s t-test with P values < 0.05 considered statistically significant.

Correlations between the number of mapped reads and the load of bacteria in blood, kidney or liver were analyzed by the Pearson correlation test. Reproducibility of the Tn-Seq experiment was assessed using the Spearman correlation coefficient. Comparison of survival curves was performed using the Mantel-Cox log rank test. Comparison of motility (diameter of growth) between strains was performed using the Student’s t test with P values < 0.05 considered statistically significant. For all statistical tests, the number of asterisks indicates the significance level; * P < 0.05, ** P < 0.01 and *** P < 0.001.

### Quantification of the bacterial load in tissues by qPCR

The number of bacteria in each sample (Input pool, serum, kidney and liver) was quantified with the Bio-Rad iQ5 real time system using the iTaq universal probe supermix. The *lipL32* gene was amplified using the LipL32-45F and LipL32-286R primers and the LipL32-189P probe as previously described [30, 39] (Table 2). The PCR mixture contained 250 nM of each primer, 150 nM of the specific probe, and 5 μl of DNA in a total volume of 20 μl. The amplification protocol consisted of 10 min at 95°C, followed by 40 cycles of amplification (95°C for 15 s and 60°C for 1 min). A negative result was assigned where no amplification occurred or if the threshold cycle (CT) was greater than 36. Real-time PCR was performed in duplicate for each sample. Results were expressed as the number of leptospires/g of tissue used for DNA extraction or number of leptospires/mL of serum or culture.

### Western Blots

*loa22*::Tn, *flaA1*::Tn, *ligB*::Tn and *lic20111*::Tn and the WT strain were grown at 30°C in EMJH, supplemented with Km when necessary, to an OD_420nm_ of ≈ 0.2. Half of the *ligB* mutant and WT cultures were incubated with 120 mM NaCl for 4 hours at 30°C to maximize *ligB* expression [40].

Samples were separated on a 4–12% gradient NuPAGE Bis Tris precast gel (Invitrogen) and transferred to a PVDF membrane (Millipore) by semi-dry transfer at 25 V for 45 min with a Bio-Rad Trans-blot Semi-dry Transfer Cell unit.

Membranes containing the *loa22* mutant were probed with a 1/1,000 dilution of Loa22 rabbit polyclonal antiserum [41] and a 1/10,000 dilution of LipL41 rabbit polyclonal antiserum [42]. Membranes containing the *flaA1* mutant were incubated with FlaA1 rabbit polyclonal antiserum at the dilution of 1/2,000 [43] and ImpL63 polyclonal antiserum at the dilution of 1/5,000 [44]. Membranes containing *ligB* mutant or *lic20111* mutant were probed with mixture of a 1/2,000 dilution of LigAB rabbit polyclonal antiserum [45] and a 1/10,000 dilution of LipL41 rabbit polyclonal antiserum as a loading control. All membranes were then incubated in 1/5,000 dilution of horseradish peroxidase-conjugated donkey anti-rabbit immunoglobulin G (Amersham) and developed by enhanced chemiluminescence (Pierce ECL reagent, Pierce).

### Motility assay

The motility of the *flaA1* and *lic20111* mutants in liquid EMJH+Km medium was analyzed by dark field microscopy and compared to the motility of the WT strain. Motility was also evaluated by spotting 0.5% agar semi-solid EMJH plates with 5 μl of four different cultures of the same mutant grown at the OD_420nm_ of 0.2. Plates were incubated for 15 days at 30°C, and diameters of growth were measured. Assays were performed in triplicate.

### Growth curves

The *lic12327a*, *lic12327b*, *lic20111*, *loa22*, *ligB* and *flaA1* mutants and WT strain were cultured at 30°C in EMJH, supplemented with Km as appropriate. Growth was monitored daily by measurements of the optical density at 420 nm on a Pharmacia Ultrospec 2000 spectrophotometer. At least three independent growth curves were performed for each mutant and strain.

### RNA extraction and genes expression

The *lic20111* mutant and WT strain were grown at 30°C to an OD_420nm_ of ≈ 0.3. Strains were cultured in duplicate. RNA was extracted from 20 mL of culture with Trizol reagent (Invitrogen) according to the manufacturer’s guidelines. Contaminating DNA was removed from RNA preparations using Turbo DNase from Ambion, and RNA was subsequently purified using the RNeasy kit (Qiagen, Valencia, CA). 1 μg of each RNA sample was converted into cDNA with iScript Reverse Transcriptase Supermix (Bio-Rad) following the manufacturer’s instructions. The amounts of specific cDNA were determined by quantitative PCR using the Bio-Rad iQ5 real time system with the iQ SYBR Green Supermix as described [46]. Primer sequences are shown in Table 2. The amount of cDNA of interest measured in each PCR assay was normalized to the amount of *rpoB* cDNA or *flaB* cDNA. The fold change of each gene was determined by the 2^-ΔΔCt^ method [47].

## Results

### Selection of the mutants

We created a library of over 800 *Himar1* transposon mutants in *L*. *interrogans* serovar Manilae strain L495. The insertion site in each mutant was determined individually by sequencing nested PCR products obtained by amplifying across one end of the transposon, as previously described by Slamti *et al*. [28].

An input pool of 42 mutants with insertions in 33 ORFs and 6 intergenic regions (Table 1) was selected to validate the Tn-Seq approach and to examine changes in the composition of the mutant population across various tissues during infection. For validation of the Tn-Seq approach, the input pool included control mutants with transposon insertions in genes that have been tested for virulence in a rodent model of lethal leptospirosis. Insertional mutations previously shown to attenuate virulence occurred in *loa22*, encoding a protein with an OmpA domain, and *lic20111/lb139*, encoding a potential phosphatase that may modulate a two-partner switch mechanism controlling an alternative sigma subunit [11, 12]. We also included mutants with insertions in genes previously shown to not be required for virulence including the adhesin gene *ligB* and the flagellar gene *flaA1* [13, 48]. Six intergenic mutants were included that harbored the transposon between open reading frames.

To identify novel *L*. *interrogans* genes required for *in vivo* fitness, the pool included 23 mutants with insertions in 20 genes encoding putative signal transduction proteins. Our rationale for focusing on this gene category was that disruptions of signaling genes would be more likely to affect *in vivo* fitness due to their potential downstream effects on multiple functions. The input pool included two mutants with insertions in *lic12324* encoding a gene containing a phosphatase domain, seven mutants with insertions in adenylate/guanylate cyclase genes, three in diguanylate cyclase or phosphodiesterase genes, five in histidine kinase genes, one in a gene encoding a member of the AcrR family of transcriptional regulators, and one in a gene encoding an alternative sigma factor (Table 1). The *lic12324*, *lic12327* and *lic12627* genes were each represented by two mutants with insertions in different locations within the same open reading frame. We also selected ten additional mutants with potential roles in *in vivo* fitness, including mutants with insertions in *lolD*, encoding a homolog of the ATPase component of the lipoprotein export system, a heme oxygenase and the flagellar gene *flaB2* (Table 1).

### The Tn-Seq experiment

Eight hamsters were challenged with the pool of 42 mutants. Four days post-challenge, blood, kidney and liver were collected. DNA extracted from these tissues and the input pool were analyzed by Illumina sequencing. 2 x 10^4^–3 x 10^6^ reads were obtained for each organ (S1 Table). On average approximately 25% of the reads were discarded from the analysis during the cleaning phase of the sequence analysis. The remaining reads were mapped to the high-quality sequence of the Fiocruz L1-130 genome using Bowtie (in the Galaxy software), and the frequency of each mutant within the bacterial population in each tissue and in each animal was determined. The nucleotide sequence of the Manilae L495 ORFs disrupted by the *Himar1* element are 98.5–100% identical with those of the corresponding ORFs in the Fiocruz L1-130 strain.

**Fig 1:**
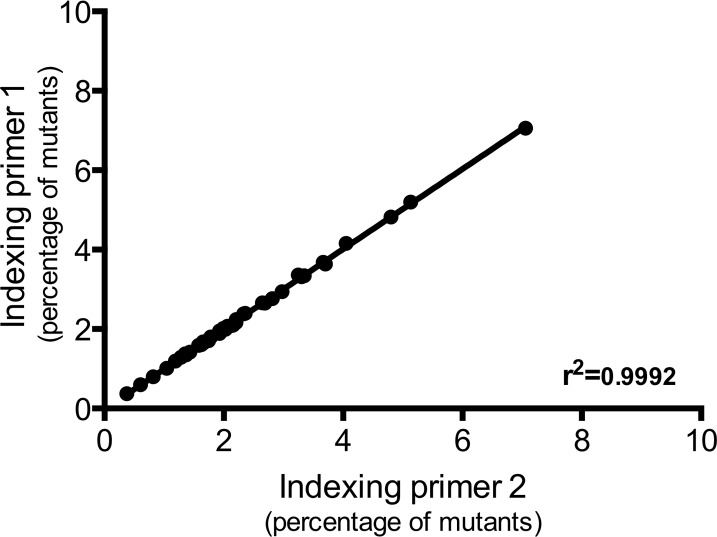
Reproducibility of the Tn-Seq experiment. We amplified the input pool DNA with two different indexing primers and sequenced both libraries with the Illumina HiSeq 2500 platform. Both sets of reads were processed as independent samples. We compared the percentage of each mutant obtained for both libraries and determined the correlation coefficient between the two libraries (r^2^ = 0.9992).

To examine the reproducibility of our Tn-Seq protocol (sample preparation and sequencing), technical replicates with the input pool DNA were performed. Two sequencing libraries were created from the DNA with two different indexing primers. A strong correlation (r^2^ = 0.9992) was observed between the composition of the population of mutants of these libraries (Fig 1), demonstrating the reproducibility of the amplification and sequencing methods.

The same DNA preparations used for Tn-Seq were also used to quantify the total number of bacteria in each sample by TaqMan qPCR targeting the *lipL32* gene. The results were expressed in terms of number of leptospires per gram of tissue or mL of serum (S1 Table). The leptospiral load in the liver, ranging from 3 x 10^4^/g to 2 x 10^8^/g, was always higher than in the kidneys, where it ranged from 2 x 10^4^/g to 1 x 10^7^/g. In serum, the number of leptospires was lower: 1 x 10^4^/mL to 3 x 10^5^/mL.

**Fig 2:**
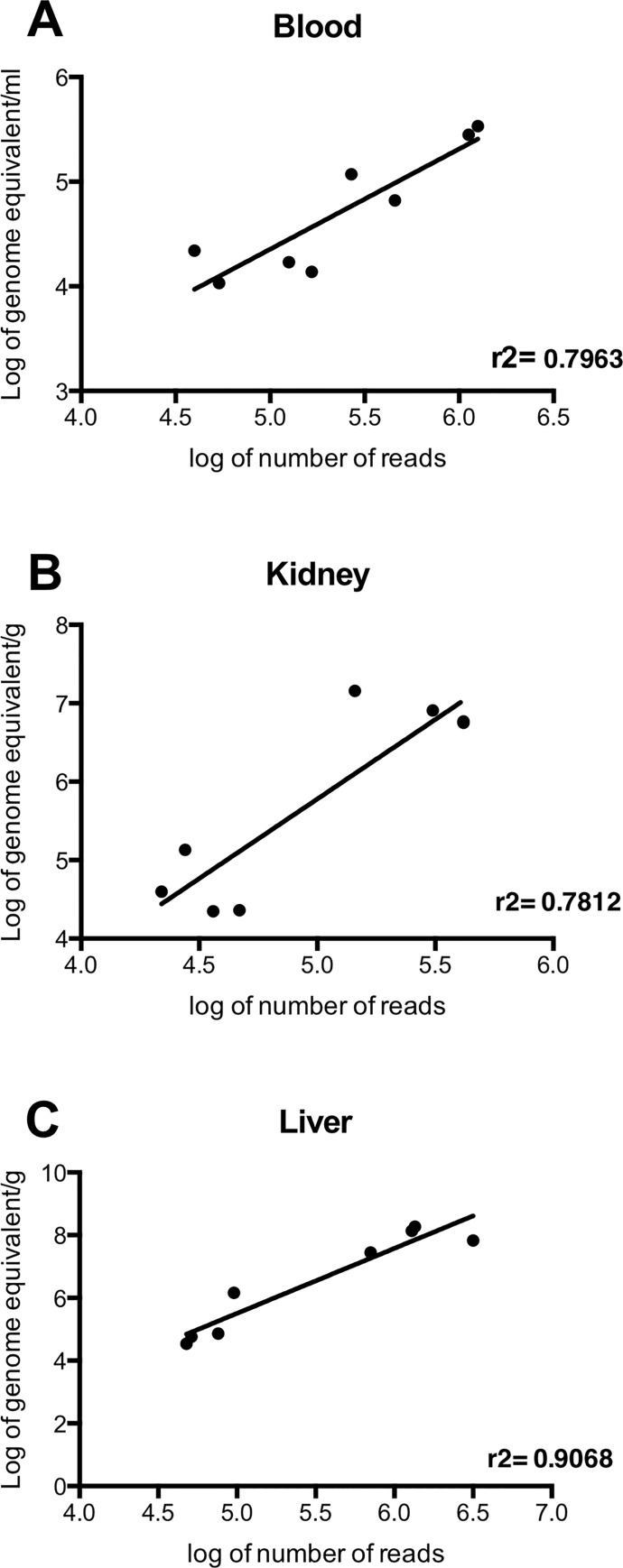
Relationship between the number of mapped reads and the bacterial load. Correlation between the log of the total number of mapped reads and the log of the number of bacteria (A) in blood, (B) kidney and (C) liver. In all tissues, there is a significant correlation between the number of reads and the number of bacteria, r^2^ = 0.7963 (P = 0.0029), r^2^ = 0.7818 (P = 0.0036) and r^2^ = 0.9068 (P = 0.0003) in blood, kidney and liver, respectively.

A significant positive correlation was found between the number of reads mapped and the burden of leptospires: r^2^ = 0.7963 (P = 0.0029), r^2^ = 0.7818 (P = 0.0036) and r^2^ = 0.9068 (P = 0.0003) in blood, kidney and liver, respectively (Fig 2A–2C).

### Changes in the composition of the population of mutants at 4 days post-challenge

Four days post-challenge, the composition of the population of mutants was quantified in blood, kidney and liver and was compared to that of the inoculum. The frequency of all mutants in the input pool was calculated, as well as their frequencies in each tissue and animal. The frequency of each mutant in the input pool ranged from 0.4% to 7% (S2 Table). All mutants were detected in all tissues with changes in the composition of the populations. The means of the percentages in blood, kidney and liver ranged from 0.2% to 8%, 0.2% to 9% and 0.2% to 13%, respectively (S2 Table).

**Fig 3:**
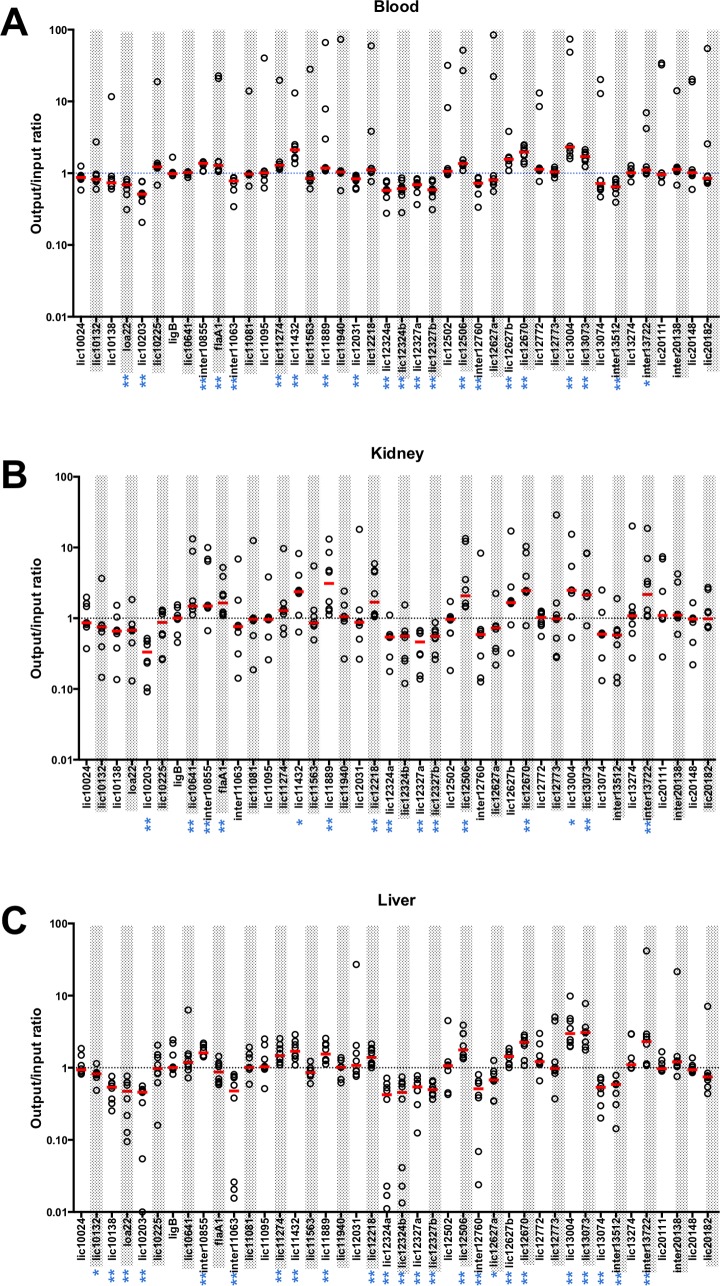
Fitness of 42 *L*. *interrogans* mutants during acute infection of hamsters. The 42 transposon mutants were grown independently, quantified, diluted at the concentration of 10^6^/mL and pooled together (input pool). Eight hamsters were challenged intraperitoneally with 1 mL of the pool of mutants. Four days post-challenge, animals were sacrificed, and blood, kidney and liver were collected (output pools). The frequency of each of the 42 mutants in the input pool and output pools was quantified using the Illumina HiSeq 2500. The output/input ratio was determined for each mutant in (A) blood, (B) kidney and (C) liver in each animal. Each ratio is represented by a white circle. For each mutant and each tissue, the median of ratios (red line) was determined and compared to 1.0 using the Wilcoxon rank test. The dotted line represents fitness of 1.0, which means no change in fitness. Mutants whose fitness is significantly affected are marked by asterisks: * P < 0.05; ** P < 0.01.

For each mutant, we calculated the output/input ratio, defined as the frequency of a mutant in the blood, kidney or liver divided by its frequency in the input pool. The output/input ratios across the 42 mutants were normalized to a median ratio to 1.0 in each animal. For each mutant and each tissue, we determined the median of the normalized ratios of the eight animals and compared it to 1.0 using the Wilcoxon signed-rank test. A fitness value of 1.0 is neutral, less than 1.0 is disadvantageous and greater than 1.0 is advantageous [49]. We observed statistically significant changes in fitness for 21, 15 and 24 mutants in blood, kidney and liver, respectively (Fig 3A–3C and S3 Table). In all tissues, eleven mutants had fitness values higher than 1.0. In contrast, ten, four and thirteen mutants had decreased fitness in the blood, kidney and liver, respectively. A total of 12 mutants had statistically significant changes in all three tissues; with either decreased (e.g., the *lic12327* and *lic10203* mutants) or increased (e.g., the *lic12506* and *lic13004* mutants) fitness.

### Control mutants

**Fig 4:**
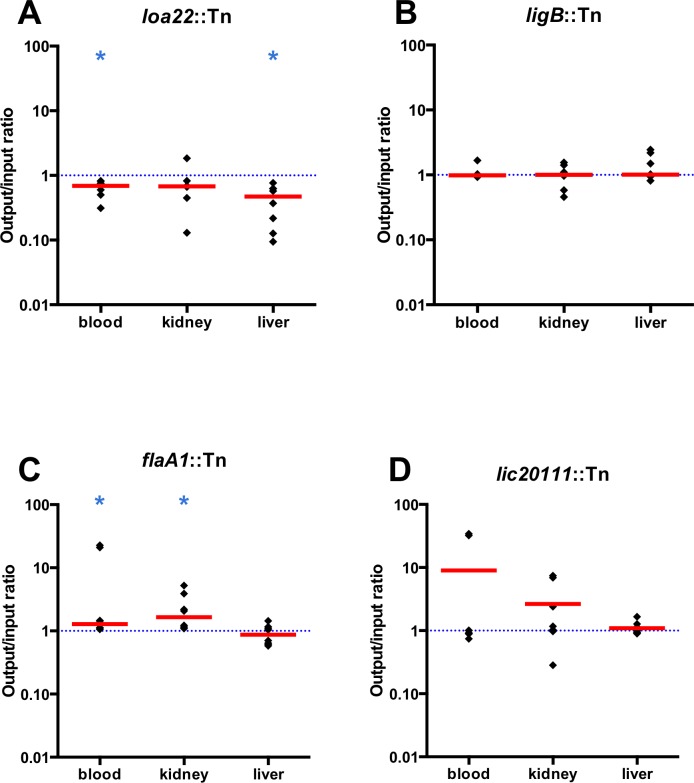
Fitness of *L*. *interrogans* control mutants during acute infection of hamsters. In the pool of mutants used to challenge the animals, we included mutants whose virulence is known to be affected: (A) *loa22*::Tn and (D) *lic20111*::Tn or unaffected: (B) *ligB*::Tn and (C) *flaA1*::Tn. The output/input ratio of each mutant was determined for each animal in blood, kidney and liver. Each ratio is represented by a black diamond and the median of these ratios by a red line. The dotted line represents fitness of 1.0, which means neutral fitness. The median was compared to 1 using the Wilcoxon rank test. The number of asterisks indicates the significance level: * P < 0.05.

**Fig 5:**
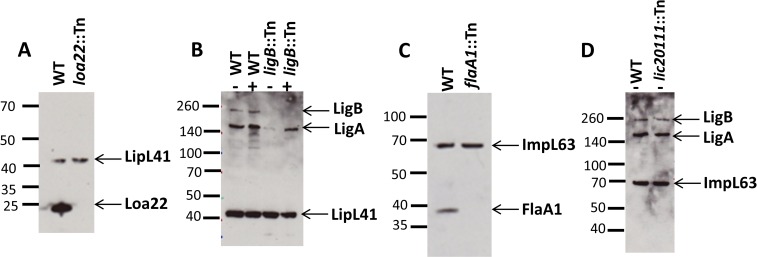
Western blots of *L*. *interrogans* control mutants. (A) Analysis of *loa22* expression by Western blotting of the *loa22* mutant and the WT strain. Blots were probed with anti Loa22 antisera. (B-D) Western blot of LigA and LigB expression in the *lic20111* mutant, *ligB* mutant and the WT strain, supplemented (+) or not (-) with 120 mM sodium chloride. Whole-cell lysates were analyzed by immunoblotting with anti-Ligs antiserum. (C) Analysis of *flaA1* expression by immunoblotting in the *flaA1* mutant and the wildtype strain (WT). Detection was performed with anti FlaA1 antisera. Anti-ImpL63 and anti-LipL41 antisera were used as a loading control. Positions of molecular mass standards are shown in kilodaltons.

**Fig 6:**
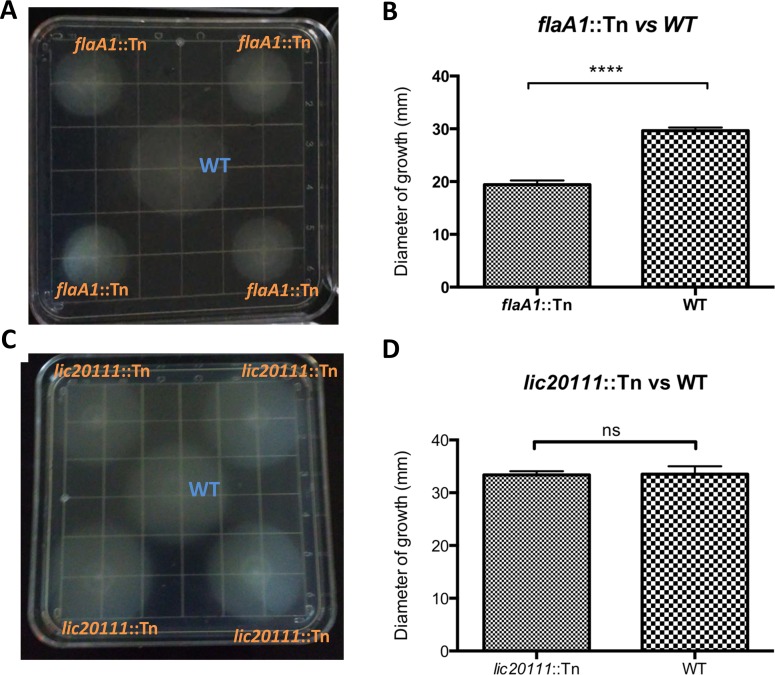
Motility assay of *L*. *interrogans* control mutants. 0.5% agar EMJH plates were spotted with the wild type strain and four different cultures of (A) the *flaA1* mutant or (C) the *lic20111* mutant. (B-D) Growth diameter of each strain was measured after two weeks incubation at 30°C. Three independent experiments were performed. A representative plate is shown from one of them.

**Fig 7:**
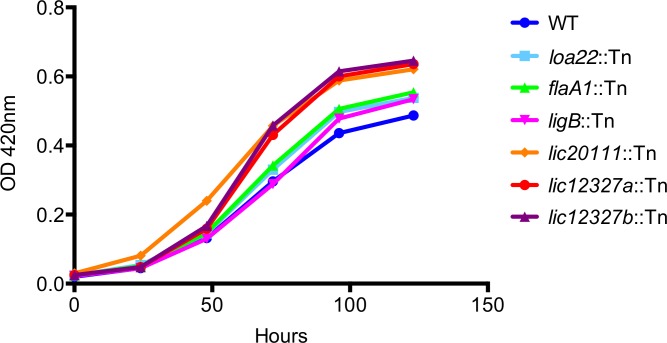
Growth curves of selected *L*. *interrogans* mutants. The *loa22*::Tn (light blue), *ligB*::Tn (pink), *flaA1*::Tn (green), *lic20111*::Tn (orange), *lic12327a*::Tn (red), *lic12327b*::Tn (purple) and the WT (dark blue) strain were cultured at 30°C in EMJH, supplemented with Km as appropriate. Growth was monitored by measuring the OD_420nm_. No defect in growth was observed for any of the strains. Data are representative of those from three independent experiments.

**Fig 8:**
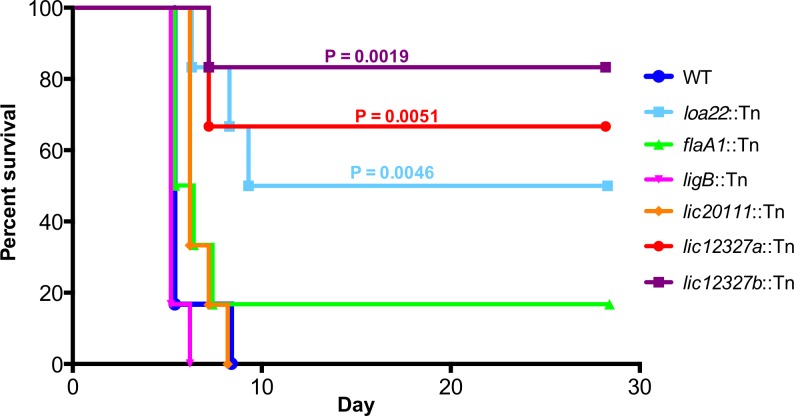
Survival experiment of hamsters infected with individual *L*. *interrogans* mutants. Hamsters were challenged IP with 10^6^ WT (dark blue), *loa22*::Tn (light blue), *ligB*::Tn (pink), *flaA1*::Tn (green), *lic20111*::Tn (orange), *lic12327a*::Tn (red) or *lic12327b*::Tn (purple) and were monitored for 28 days or until they met endpoint criteria. All animals infected with the WT strain, *ligB*::Tn and *lic20111*::Tn met the endpoint criteria in 5 to 8 days (*ligB*::Tn and *lic20111*::Tn survival curves not statistically different to the WT, P = 0.5514 and P = 0.1557, respectively). One animal challenged with *flaA1*::Tn survived, but the survival curve did not differ from the WT (P = 0.4364). Three hamsters infected with *loa22*::Tn met the endpoint criteria in 6 to 9 days, survival curve statistically different from the WT (P = 0.0046). Two animals challenged with *lic12327a*::Tn and one with the *lic12327b* mutant met the endpoint criteria in 7 days. Both *lic12327* mutants showed a statistically different survival curve than the WT, P = 0.0051 and P = 0.019 respectively.

In addition to the Tn-Seq results (Fig 4), we conducted Western blots (Fig 5), motility assays (Fig 6) and growth curve analysis (Fig 7) to confirm previously described phenotypes of the control mutants. To determine whether the virulence of the control mutants was as observed in previous studies, we assessed their virulence in a survival experiment (Fig 8 and S4 Table) and examined kidney colonization. Growth curves showed that none of the control mutants exhibited a defect in *in vitro* growth compared to the WT strain (Fig 7).

#### *loa22* mutant

At four days post-challenge, the fitness of the *loa22* mutant was decreased in all tissues (P = 0.0078 for both blood and liver) (Fig 4A and S3 Table). Immunoblots confirmed the lack of production of Loa22 in the *loa22* mutant. The protein was only detected in the WT strain (Fig 5A). We also confirmed the virulence phenotype by challenging groups of 6 hamsters with 10^6^ *loa22* mutant or with the WT. All animals infected with the WT strain died between 5 to 8 days post infection whereas only 3 hamsters challenged with *loa22* mutant met the endpoint criteria (P = 0.0046; Fig 8 and S4 Table). All kidneys cultured from animals infected with the WT strain or *loa22* mutant were culture positive in EMJH.

#### *ligB* mutant

In the Tn-Seq experiment, the fitness of the *ligB* mutant was not affected (Fig 4B and S3 Table) in any of the tissues. We confirmed by immunoblot (Fig 5B) that the *ligB* mutant did not produce LigB, whether or not the medium was supplemented with NaCl. Although LigB was not produced, LigA was produced by the *ligB* mutant. We challenged hamsters with 10^6^ of the *ligB* mutant or WT strain and did not observe differences in survival (P = 0.5514; Fig 8 and S4 Table) and kidneys from all animals were culture positive.

#### *flaA1* mutant

The fitness of *flaA1* mutant in the Tn-Seq experiment was significantly higher than 1.0 in blood and kidney (P = 0.0078; Fig 4C). Western blots with the *flaA1* mutant confirmed that the mutant did not produce FlaA1 (Fig 5C). Motility was decreased compared to the wild-type strain as observed in the darkfield microscope. The diameter of growth of the *flaA1* mutant on the motility plate was smaller than that of the wildtype (P = 0.0002; Fig 6A and 6B). We challenged hamsters with 10^6^ *flaA1* mutant or the WT strain and we did not find any difference in survival (Fig 8 and S4 Table) and kidneys from all animals were culture positive.

#### *lic20111* mutant

The fitness of the *lic20111* mutant was not affected in the Tn-Seq experiment (Fig 4D and S3 Table). Hamsters were challenged intraperitoneally with 10^6^ WT or 10^6^ *lic20111* mutants cells and monitored daily until they met the endpoint criteria or for 21 days. All animals infected with the WT strain or with *lic20111*::Tn met the endpoint criteria within 5–8 days. The survival curves did not differ significantly between the strains (Fig 8 and S4 Table). The motility assay on 0.5% agar EMJH plates showed no difference in the diameter of growth of the *lic20111* mutant compared to the WT (Fig 6C and 6D). The *lic20111* mutant produced similar amounts of LigB and LigA as the wildtype (Fig 5D). We examined the transcript levels of the downstream genes in the *lic20111* mutant and the WT strain by qRT-PCR. When *rpoB* was used as the reference gene, the *lic20111* mutant exhibited 1.6-, 1.4- and 2.5-fold drops for *lic20110*, *lic20109* and *lic20108* RNA respectively, relative to wild-type strain. When *flaB* was used as the reference gene, the same transcripts in the *lic20111* mutant exhibited 1.1-, 0.9- and 1.6-fold reductions.

### Pairs of mutants with transposon insertions in the same gene

**Fig 9:**
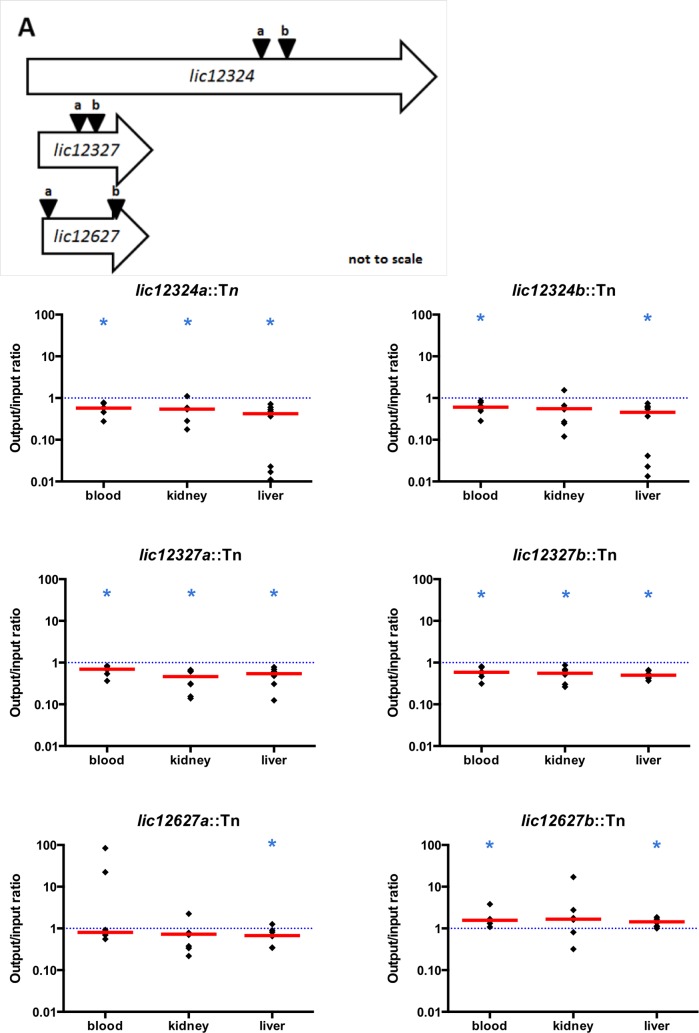
Pairs of mutants with transposon insertions in the same gene. (A) Locations of transposon insertion sites. In the pool of mutants used to challenge the animals, we included three pairs of mutants that have insertion sites at different locations in the same gene. The location of both transposon insertions is designated by black arrows in each of the following genes: *lic12324* (insertion sites: 2806250 and 2806718 in the Fiocruz L1-130 genome), *lic12327* (insertion sites 2810027 and 2810271) and *lic12627* (insertion sites: 3177595 and 3178505). (B-G) Fitness of each pair of mutants. The output/input ratios for each of the eight animals were determined for each mutant in blood, kidney and liver. Each ratio is represented by a black diamonds and the median of these ratios by a red line. The dotted line represents fitness of 1.0, which means neutral fitness. The median was compared to 1.0 using the Wilcoxon rank test. The number of asterisks indicates the significance level: * P < 0.05. No statistically significant differences were observed between the pair of *lic12327* mutants or between the *lic12324* mutants for any tissue. The comparison of the ratios of these two *lic12627* mutants confirmed statistically significant differences in kidney (P = 0.0379) and liver (P = 0.0011).

In the pool of mutants used to challenge the animals, we included three pairs of mutants with different transposon insertion sites in the same gene: *lic12324*, *lic12327* and *lic12627*. The insertion sites in the *lic12324* gene were separated by 468 bp and by only 244 bp in the *lic12327* gene. In the *lic12627* gene insertions sites were farther apart (separated by 910 bp) (Fig 9A).

For the paired *lic12324* mutants and paired *lic12327* mutants, we observed a statistically significant decrease of *in vivo* fitness in both members of the pairs (Fig 9B–9E). When we compared *lic12324a*::Tn to *lic12324b*::Tn and *lic12327a*::Tn to *lic12327b*::Tn, we did not see any statistically significant differences in their fitness.

In the third pair of mutants, the fitness of the *lic12627a* mutant (insertion site in the 5’ end of the gene) was reduced in liver and kidney but significantly only in liver (Fig 9F and 9G). However, the fitness of the *lic12627b* mutant (insertion site in the 3’ end of the gene) was significantly higher than 1.0 in blood and liver. The comparison of the ratios of these two mutants confirmed statistically significant differences in kidney (P = 0.0379) and liver (P = 0.011).

Previously, expression of the *lic12327* gene has been shown to be upregulated by osmolarity [50], suggesting its role in dissemination and survival in the host. Because of the decrease in fitness in both *lic12327* mutants, we confirmed that neither mutant had a growth defect compared to the WT strain (Fig 7). We studied their virulence separately by challenging hamsters IP with 10^6^ leptospires. Only one of six and two of six animals infected with *lic12327b* and *lic12327a* mutants, respectively, met endpoint criteria (survival curves statistically different from the WT, P = 0.0019 and P = 0.0051, respectively; Fig 8 and S4 Table). All animals had kidney colonization whether or not they met endpoint criteria.

### Signal transduction genes

Twenty-three mutants with transposon insertions in putative signal transduction genes were included in this study (Table 1). Seven mutants did not present any change in any tissue. The sixteen other mutants exhibited *in vivo* fitness that differed significantly from 1.0: five mutants had changes in only one tissue, three in two tissues and eight in all three tissues (Fig 3 and S3 Table). Among the eight mutants with changes in fitness in all tissues, five presented an increase in their fitness whereas three showed a decrease (Fig 3).

The five mutants with an increase in their fitness in all tissues had transposon insertions in adenylate or guanylate cyclase genes (*lic12506*::Tn, *lic12670*::Tn and *lic13004*::Tn), in a histidine kinase gene (*lic11432*::Tn) or in a transcriptional regulator gene (*lic13073*::Tn) (Fig 3 and S3 Table). The three mutants with decreased fitness in all tissues had transposon insertions in an adenylate or guanylate cyclase gene (*lic12327a*::Tn and *lic12327b*::Tn) or in a phosphatase gene (*lic12324a*::Tn) (Fig 3 and S3 Table).

### Non signal transduction genes

Thirteen mutants with transposon insertions in non-signaling genes were included in the pool of mutants (Fig 3 and S3 Table). Three of these were “control” mutants and their behavior has been described above. Among the ten other mutants, *lic11889*::Tn, which has a transposon inserted in a flagellar protein, had increased fitness in all tissues and *lic10203*::Tn, which has an insertion in an epimerase gene, had diminished fitness. The fitness of the eight remaining mutants was not affected in any tissue.

### Intergenic regions

Six mutants with transposon insertions in intergenic regions were included in the input pool. Only one (*inter20138*::Tn) did not exhibit a change in fitness; two mutants (*inter10855*::Tn and *inter13722*::Tn) had increased in fitness in all tissues (Fig 3 and S3 Table). The three other mutants showed decreased fitness in blood and liver.

### Distribution of mutant fitness values in each animal

The distribution of the fitness values of the mutants differed among animals. We identified two types of distribution: a narrow distribution where all mutants have output/input ratios ranging from less than 2.5 log and a broad distribution where the range of ratios varies from more than 2.5 log (S2 Fig). In blood and kidney, these two distributions are observed in the same animals: narrow distribution in animals 3, 5, 6 and 7, broad distribution in animals 1, 2, 4 and 8 (S2A and S2B Fig). In liver, only two animals have a narrow distribution, animal 3 and animal 7 (S2C Fig).

## Discussion

We have developed a Tn-Seq assay to identify *L*. *interrogans* virulence genes candidates. The combination of transposon mutagenesis with the power of high-throughput sequencing successfully detected mutants with *in vivo* fitness defects. A major advantage of Tn-Seq is the ability to screen a large pool of mutants for altered *in vivo* fitness with a limited number of animals. This approach allowed us to reduce the cost of such an extensive screening of mutants, first by using a small number of animals and second by performing high throughput sequencing in a single lane in the Illumina system. Our findings with a small number of mutants suggest that Tn-Seq can be used as a first step to identify virulence genes of *L*. *interrogans* by screening large pools of mutants for defects in *in vivo* fitness. However, because not all *L*. *interrogans* mutants with diminished fitness within a pool of mutants will be attenuated in virulence [15], experiments with larger numbers of mutants need to be done to better understand the relationship between fitness and virulence.

This was the first Tn-Seq experiment performed with leptospiral mutants to assess their *in vivo* fitness. Due to the high bacterial tissue load, we were able to obtain sequencing reads from the DNA extracted directly from blood, kidney and liver. A culture step prior to DNA extraction was not necessary and allowed us to avoid *in vitro* growth bias. However, the approach can overestimate the relative abundance of a mutant by measuring the DNA from dead bacteria. We expect the contribution from dead leptospires to be minimal due to the exponential growth of the bacteria in tissues during the four days of infection [39].

We demonstrated the reproducibility of our protocol by processing the input pool DNA with two different indexing primers and comparing the frequency of each mutant obtained in both libraries (Fig 1). A strong correlation was observed between these libraries, demonstrating the reproducibility of our protocol (PCR and sequencing). To validate Tn-Seq as a method to identify virulence genes of *L*. *interrogans* and to examine the relationship of fitness to virulence, the input pool included mutants with insertions in genes whose virulence had been examined in earlier studies. Transposon insertions in *loa22* and *lic20111* attenuated virulence [11, 12], whereas a transposon insertion in *flaA1* and a targeted deletion of *ligB* had no effect [13, 48]. In our experiments, no defect in fitness of either the *ligB* or *flaA1* mutant was observed in any of the tissues tested (Fig 4B and 4C) and, as previously described, no attenuation in their virulence compared to the WT strain was seen in the hamster model (Fig 8). The *loa22* mutant exhibited a decrease in *in vivo* fitness in blood and liver (Fig 4A) and attenuation in its virulence in the animal model (Fig 8). These results confirm previous studies showing a role for *loa22* in virulence [11]. The partial virulence attenuation of the *loa22* mutant differed from the findings of the study by Ristow *et al*., in which the mutant was completely avirulent [11]. This difference may be related to the different parent strain: in our study, the *loa22* mutant was generated in the highly virulent L495 strain (LD_50_ < 10^2^) [51] whereas it was previously obtained in the less virulent Lai strain 56601 (LD_50_ > 10^7^) [11].

Surprisingly, neither the *in vivo* fitness (Fig 4D) nor the virulence (Fig 8) of the *lic20111* mutant was affected, in contrast to the phenotype of another *lic20111* mutant described in an earlier study [12]. The *in vitro* phenotype of our *lic20111* mutant was similar to the WT in its motility (Fig 6C and 6D), Lig protein production (Fig 5D) and growth (Fig 7), which is in opposition to the loss of virulence, reduction in motility, and diminished *lig* transcript production observed with an *lic20111* mutant described by Eshghi *et al*. In that study, it was proposed that *lic20111* is the first gene of a five-gene operon and that the insertion of the transposon in *lic20111* caused attenuation of its virulence in the hamster model by polar effects on downstream transcription, which was verified by qRT-PCR [12]. We also observed diminished transcription of the downstream genes with our *lic20111* mutant, although the effect was weak. The transposon is inserted in different locations in the *lic20111* mutants: at the 3’ end of the gene in the Eshghi *et al*. study and near the middle of the gene in our study (S1 Fig). The difference in virulence, motility, and *lig* expression between the two *lic20111* mutants suggests that the location of the transposon within a gene may influence the mutant’s phenotype. By extension, the difference in *in vivo* fitness observed between the two *lic12627* mutants (Fig 9F) may be explained by the different insertion sites of the transposon (Fig 9A). This would suggest that the nearly full length LIC12627 protein generated from the *lic12627b* mutant, which harbors the transposon close to the 3’ end of *lic12327*, retains adequate function to maintain the fitness of the strain. Although we lack experimental data that confirms expression of an active gene product from the *lic12627b* mutant, a similar effect was proposed by Lin *et al*., [18, 52], who noted that in their collection of 4,479 transposon insertion mutants in *B*. *burgdorferi*, insertions in the last 10% of ORFs were over-represented.

Five of the mutants included in our study have been studied previously in the experiment reported by Marcsisin *et al*. [15], in which each animal was inoculated with a pool of 10 mutants. Similar results were obtained with some of our mutants. For instance, the *lic12324* mutant, whose *in vivo* fitness was decreased in all tissues in our study, was detected by standard PCR in kidney and blood from only one and two animals out of five, respectively, in Marcsisin’s study. Comparable results were obtained in both studies with the *lic13274* mutant, which was detected from four out of five animals in Marcsisin’s study and for which no change in fitness was observed in our Tn-Seq experiment. In contrast, while we observed no change or an increase in *in vivo* fitness of our *lic20182* and *lic10641* mutants (Fig 3A–3C), these mutants could not be detected in blood and kidney (except in the blood of one animal with *lic10641*::Tn) in Marcsisin’s study [15]. These differences could result from differences in the inoculation dose, time of infection, or an unrecognized shortcoming with our assay.

We identified two *lic12327* mutants with reduced *in vivo* fitness in blood, kidney and liver (Fig 9D and 9E). *lic12327* encodes a putative adenylate/guanylate cyclase that contains a GAF domain and an adenylate/guanylate cyclase catalytic domain. This gene has been shown to be upregulated by physiological osmolarity [50] suggesting a role during host infection. The transposon insertion sites are 244 nucleotides apart from each other in these two mutants but both are located in the GAF domain. Both mutants were attenuated for virulence when tested individually in the hamster model (Fig 8 and S4 Table) and were recovered from kidneys. Experiments from another study demonstrated that a putative adenylate cyclase secreted from *L*. *interrogans* (LA4008/LIC13201) elevated cAMP levels in a human monocytic cell line [53]. These observations suggest that cyclic nucleotides produced by *L*. *interrogans* play a variety of roles during infection.

In contrast, we observed an increase in fitness for several mutants. The advantage of losing functional genes has been observed with *Salmonella enterica*, in which 25% of spontaneous deletions caused by randomly-inserted transposons caused enhanced growth rates under at least one of three growth conditions [54]. The enhanced fitness of mutants could be explained in part by the reduced metabolic burden in mutants that no longer synthesize proteins that are not essential for growth [54]. Alternatively, the functions provided by the disrupted genes may be provided by nearby mutants in the pool through the production of extracellular “common goods” [55]. For example, an inactivating mutation in the *Pseudomonas aeruginosa* gene encoding the quorum sensing regulator LasR results in the mutant out-competing the wild-type strain during co-culture [56]. Because signaling proteins are more likely that structural proteins to affect expression of multiple genes, our pool of mutants may be enriched for those with increase fitness *in vivo*.

In addition to the control mutants that have previously been described, we included intergenic mutants in the input pool as potential controls for neutral effects on *in vivo* fitness. Surprisingly, only one out of six intergenic mutants was unaffected in fitness. Two of the intergenic mutants had an increase in fitness and three others exhibited a decrease (Fig 3A–3C). These unexpected results can be due to the insertion of the transposon into a small RNA gene, into a promoter, regulatory or transcriptional terminator region, or into a protein-coding gene that has not been annotated. None of the small RNA genes identified in Camaino *et al*.’s RNA-seq study were disrupted in our set of mutants [57].

Nevertheless, there are several general limitations to Tn-Seq that need to be considered. A decrease in fitness might be due to a mutant being out-competed by the other mutants in the pool rather than a direct effect of the transposon insertion on fitness [58, 59]. An absence of change in *in vivo* fitness of a mutant that otherwise would have poor fitness in individual infections could be due to cooperation between mutants [60, 61]. Indeed, 325 exoproteins have been identified in *L*. *interrogans* cultured under conditions mimicking infection [62]. Therefore, a mutant that fails to produce one of these exoproteins could be complemented intercellularly by another mutant. Additionally, bottlenecks during infection may impede recovery of random mutants from the original pool. However, all mutants from the input pool were recovered in all three tissues suggesting that bottlenecks did not significantly affect our experiment. Nevertheless, increasing the size of the pool may cause the stochastic loss of mutants during infection. This can be minimized by increasing the size of the inoculum [17]. Despite these limitations, we anticipate that Tn-Seq can be used to screen larger pools of *L*. *interrogans* mutants in a limited number of animals.

## Supporting Information

S1 Table Number of reads mapped to the Fiocruz L1-130 genome and number of genome equivalents (Geq) per milliliter of serum or gram of kidney and liver. (DOCX)

S2 Table Frequencies of each mutant in the input pool, in blood, kidney and liver of each animal.Mean of the frequencies per mutants and standard deviation. (DOCX)

S3 Table Output/input ratios of each mutant frequencies in each animal in blood, kidney and liver. Median of ratios and variance per mutant.
(DOCX)

S4 Table Survival experiment with selected mutants. (DOCX)

S1 Fig Insertion sites of the transposon in *loa22*, *flaA1*, *ligB* and *lic20111*. The red arrow represents the insertion site in this study and the grey one in previously described studies: Ristow *et al*. [11], Lambert *et al*. [48] and Esghi *et al*. [12], for *loa22*::Tn, *flaA1*::Tn and *lic20111*::Tn respectively. The gray zone in *ligB* gene shows the part of the gene that was removed in Croda *et al*, study [13].
(TIFF)

S2 Fig Fitness distribution of mutants in each animal. The output/input ratio of each mutant was determined for each animal in (A) blood, (B) kidney and (C) liver. The ratio of each mutant is represented by a black diamonds and the median of these ratios by a red line.
(TIFF)
