# Supplementary material for: High-Throughput Parallel Sequencing to Measure Fitness of Leptospira interrogans Transposon Insertion Mutants during Acute Infection
Source: PLoS Negl Trop Dis. 2016 Nov 8;10(11):e0005117. doi: 10.1371/journal.pntd.0005117 (PMC5100919; doi:10.1371/journal.pntd.0005117)
Supplement: S1 Table — (DOCX) [file pntd.0005117.s001.docx]

**S1 Table.** Number of reads mapped to the Fiocruz L1-130 genome and number genome equivalent (Geq) per ml of serum or g of kidney and liver.

|  | SERUM | | KIDNEY | | LIVER | |
| --- | --- | --- | --- | --- | --- | --- |
| Animal | Number of reads | Number of Geq/ml | Number of reads | Number of Geq/g | Number of reads | Number of Geq/g |
| 1 | 4.6E+05 | 6.6E+04 | 3.1E+05 | 8.1E+06 | 7.0E+05 | 2.8E+07 |
| 2 | 2.7E+05 | 1.2E+05 | 4.1E+05 | 5.9E+06 | 1.3E+06 | 1.4E+08 |
| 3 | 3.9E+04 | 2.2E+04 | 3.6E+04 | 2.2E+04 | 7.6E+04 | 7.3E+04 |
| 4 | 1.3E+06 | 3.4E+05 | 1.4E+05 | 1.4E+07 | 3.1E+06 | 6.7E+07 |
| 5 | 1.3E+05 | 1.7E+04 | 2.8E+04 | 1.3E+05 | 9.6E+04 | 1.4E+06 |
| 6 | 5.4E+04 | 1.1E+04 | 4.7E+04 | 2.3E+04 | 4.8E+04 | 3.5E+04 |
| 7 | 1.6E+05 | 1.4E+04 | 2.2E+04 | 4.0E+04 | 5.2E+04 | 5.7E+04 |
| 8 | 1.1E+06 | 2.8E+05 | 4.1E+05 | 5.6E+06 | 1.4E+06 | 1.9E+08 |
