# Supplementary material for: High-Throughput Parallel Sequencing to Measure Fitness of Leptospira interrogans Transposon Insertion Mutants during Acute Infection
Source: PLoS Negl Trop Dis. 2016 Nov 8;10(11):e0005117. doi: 10.1371/journal.pntd.0005117 (PMC5100919; doi:10.1371/journal.pntd.0005117)
Supplement: S2 Table — (DOCX) [file pntd.0005117.s002.docx]

**S2 Table.** Frequencies of each mutant in the input pool, in blood, kidney and liver of each animal.

Mean of the frequencies per mutants and standard deviation.

| **INPUT** | | | | |
| --- | --- | --- | --- | --- |
|  | **IP1** | **IP2** | **Mean** | **Stdev** |
| **LIC10024** | 1.654 | 1.643 | 1.648 | 0.008 |
| **LIC10132** | 2.203 | 2.163 | 2.183 | 0.028 |
| **LIC10138** | 2.158 | 2.105 | 2.131 | 0.037 |
| **LIC10191** | 2.975 | 2.943 | 2.959 | 0.022 |
| **LIC10203** | 0.816 | 0.802 | 0.809 | 0.010 |
| **LIC10225** | 2.029 | 1.995 | 2.012 | 0.024 |
| **LIC10464** | 2.333 | 2.385 | 2.359 | 0.037 |
| **LIC10641** | 2.687 | 2.656 | 2.671 | 0.022 |
| **Inter10855** | 1.043 | 1.014 | 1.029 | 0.021 |
| **LIC10788** | 3.348 | 3.340 | 3.344 | 0.006 |
| **Inter11063** | 1.929 | 1.892 | 1.911 | 0.026 |
| **LIC11081** | 2.209 | 2.242 | 2.226 | 0.023 |
| **LIC11095** | 1.782 | 1.802 | 1.792 | 0.014 |
| **LIC11274** | 1.282 | 1.288 | 1.285 | 0.004 |
| **LIC11432** | 2.138 | 2.092 | 2.115 | 0.033 |
| **LIC11563** | 3.667 | 3.680 | 3.673 | 0.009 |
| **LIC11889** | 1.744 | 1.708 | 1.726 | 0.025 |
| **LIC11940** | 1.352 | 1.375 | 1.364 | 0.016 |
| **LIC12031** | 1.992 | 2.012 | 2.002 | 0.015 |
| **LIC12218** | 2.815 | 2.764 | 2.789 | 0.036 |
| **LIC12324a** | 3.304 | 3.319 | 3.311 | 0.011 |
| **LIC12324b** | 1.430 | 1.417 | 1.424 | 0.009 |
| **LIC12327a** | 2.062 | 2.071 | 2.066 | 0.006 |
| **LIC12327b** | 1.369 | 1.348 | 1.359 | 0.015 |
| **LIC12502** | 1.929 | 1.949 | 1.939 | 0.014 |
| **LIC12506** | 1.654 | 1.673 | 1.664 | 0.014 |
| **Inter12760** | 1.985 | 1.979 | 1.982 | 0.004 |
| **LIC12627a** | 4.051 | 4.156 | 4.103 | 0.074 |
| **LIC12627b** | 1.950 | 1.934 | 1.942 | 0.012 |
| **LIC12670** | 1.187 | 1.193 | 1.190 | 0.005 |
| **LIC12772** | 2.360 | 2.394 | 2.377 | 0.024 |
| **LIC12773** | 0.602 | 0.598 | 0.600 | 0.002 |
| **LIC13004** | 1.714 | 1.699 | 1.707 | 0.011 |
| **LIC13073** | 1.622 | 1.607 | 1.614 | 0.010 |
| **LIC13074** | 5.130 | 5.198 | 5.164 | 0.048 |
| **Inter13512** | 3.702 | 3.640 | 3.671 | 0.044 |
| **LIC13274** | 0.372 | 0.370 | 0.371 | 0.002 |
| **Inter13722** | 4.800 | 4.821 | 4.810 | 0.015 |
| **LIC20111** | 3.247 | 3.360 | 3.303 | 0.080 |
| **Inter20138** | 1.578 | 1.591 | 1.585 | 0.009 |
| **LIC20148** | 2.648 | 2.657 | 2.653 | 0.006 |
| **LIC20182** | 7.058 | 7.058 | 7.058 | 0.000 |
| **others** | 2.093 | 2.068 | 2.080 | 0.018 |

| BLOOD |  |  |  |  |  |  |  |  |  |  |
| --- | --- | --- | --- | --- | --- | --- | --- | --- | --- | --- |
| **Animal** | **1** | **2** | **3** | **4** | **5** | **6** | **7** | **8** | **Mean** | **SD** |
| LIC10024 | 0.351 | 0.202 | 1.443 | 0.060 | 1.489 | 1.339 | 1.496 | 0.109 | 0.811 | 0.64 |
| LIC10132 | 1.013 | 0.261 | 1.798 | 0.071 | 2.055 | 1.724 | 2.029 | 0.149 | 1.137 | 0.82 |
| LIC10138 | 4.225 | 0.200 | 1.294 | 0.060 | 1.726 | 1.410 | 1.697 | 0.220 | 1.354 | 1.26 |
| LIC10191 | 0.158 | 0.255 | 2.056 | 0.085 | 2.344 | 2.209 | 2.429 | 0.169 | 1.213 | 1.05 |
| LIC10203 | 0.029 | 0.061 | 0.433 | 0.017 | 0.602 | 0.387 | 0.615 | 0.037 | 0.272 | 0.25 |
| LIC10225 | 0.233 | 0.377 | 2.555 | 0.100 | 2.326 | 2.736 | 2.366 | 4.290 | 1.873 | 1.40 |
| LIC10464 | 0.671 | 0.334 | 2.458 | 0.096 | 2.264 | 2.261 | 2.316 | 0.248 | 1.331 | 1.01 |
| LIC10641 | 0.475 | 54.865 | 2.808 | 0.111 | 2.615 | 2.704 | 2.528 | 0.264 | 8.296 | 17.64 |
| Inter10855 | 0.186 | 0.197 | 1.489 | 10.430 | 1.404 | 1.467 | 1.330 | 0.125 | 2.079 | 3.21 |
| LIC10788 | 12.910 | 0.699 | 3.788 | 2.941 | 3.484 | 3.716 | 3.540 | 0.544 | 3.953 | 3.60 |
| Inter11063 | 0.111 | 0.216 | 1.509 | 0.057 | 1.581 | 1.471 | 1.667 | 0.126 | 0.842 | 0.72 |
| LIC11081 | 5.284 | 0.318 | 2.114 | 0.092 | 2.098 | 2.141 | 2.145 | 0.167 | 1.795 | 1.59 |
| LIC11095 | 0.190 | 0.253 | 1.709 | 3.022 | 1.816 | 1.890 | 1.920 | 0.162 | 1.370 | 0.98 |
| LIC11274 | 0.246 | 0.246 | 1.590 | 6.561 | 1.559 | 1.821 | 1.516 | 2.878 | 2.052 | 1.88 |
| LIC11432 | 0.491 | 0.712 | 5.347 | 0.186 | 3.272 | 4.460 | 3.530 | 3.144 | 2.643 | 1.82 |
| LIC11563 | 0.378 | 0.407 | 3.038 | 0.121 | 3.467 | 3.134 | 3.389 | 11.690 | 3.203 | 3.49 |
| LIC11889 | 0.878 | 16.426 | 2.028 | 0.081 | 1.860 | 2.044 | 1.860 | 1.538 | 3.339 | 4.99 |
| LIC11940 | 0.132 | 0.203 | 1.436 | 0.057 | 1.375 | 1.412 | 1.474 | 11.354 | 2.180 | 3.52 |
| LIC12031 | 0.211 | 0.247 | 1.841 | 0.067 | 1.812 | 1.609 | 1.769 | 0.136 | 0.961 | 0.80 |
| LIC12218 | 1.817 | 0.433 | 2.950 | 6.975 | 3.097 | 3.216 | 2.857 | 0.242 | 2.698 | 1.96 |
| LIC12324a | 0.156 | 0.275 | 1.886 | 0.078 | 2.386 | 1.942 | 2.588 | 0.171 | 1.185 | 1.04 |
| LIC12324b | 0.069 | 0.114 | 0.853 | 0.036 | 1.185 | 0.909 | 1.131 | 0.080 | 0.547 | 0.48 |
| LIC12327a | 0.128 | 0.200 | 1.486 | 0.058 | 1.628 | 1.505 | 1.732 | 0.127 | 0.858 | 0.73 |
| LIC12327b | 0.072 | 0.113 | 0.760 | 0.034 | 1.062 | 0.811 | 1.043 | 0.073 | 0.496 | 0.43 |
| LIC12502 | 10.490 | 0.277 | 2.031 | 0.077 | 2.184 | 2.092 | 1.981 | 1.806 | 2.617 | 3.08 |
| LIC12506 | 0.308 | 0.365 | 2.317 | 1.880 | 2.053 | 2.209 | 2.134 | 9.766 | 2.629 | 2.80 |
| Inter12760 | 0.114 | 0.208 | 1.390 | 0.062 | 1.632 | 1.410 | 1.728 | 0.116 | 0.833 | 0.72 |
| LIC12627a | 15.513 | 0.445 | 2.879 | 14.534 | 3.671 | 3.130 | 3.448 | 0.257 | 5.485 | 5.65 |
| LIC12627b | 1.258 | 0.454 | 3.302 | 0.123 | 2.816 | 3.128 | 2.567 | 0.240 | 1.736 | 1.27 |
| LIC12670 | 0.272 | 0.424 | 2.917 | 0.106 | 2.096 | 2.656 | 2.101 | 0.195 | 1.346 | 1.13 |
| LIC12772 | 0.309 | 0.378 | 2.755 | 1.310 | 2.592 | 2.765 | 2.579 | 2.290 | 1.872 | 0.98 |
| LIC12773 | 0.088 | 0.088 | 0.686 | 0.027 | 0.566 | 0.569 | 0.732 | 9.798 | 1.569 | 3.12 |
| LIC13004 | 21.413 | 0.585 | 3.502 | 22.011 | 2.901 | 3.760 | 2.723 | 9.402 | 8.287 | 8.10 |
| LIC13073 | 0.409 | 0.494 | 3.221 | 0.131 | 2.471 | 2.916 | 2.501 | 0.226 | 1.546 | 1.25 |
| LIC13074 | 11.285 | 0.457 | 3.061 | 4.379 | 3.950 | 3.303 | 4.128 | 0.273 | 3.855 | 3.18 |
| Inter13512 | 0.246 | 0.324 | 2.496 | 0.095 | 2.975 | 2.700 | 2.992 | 0.216 | 1.506 | 1.29 |
| LIC13274 | 0.061 | 0.062 | 0.481 | 0.016 | 0.346 | 0.420 | 0.379 | 0.032 | 0.225 | 0.19 |
| Inter13722 | 5.686 | 0.846 | 5.302 | 0.199 | 5.215 | 5.194 | 4.902 | 2.293 | 3.705 | 2.09 |
| LIC20111 | 0.418 | 15.318 | 3.086 | 0.127 | 3.232 | 2.896 | 3.315 | 12.854 | 5.156 | 5.32 |
| Inter20138 | 0.183 | 0.268 | 1.780 | 0.069 | 1.768 | 1.895 | 1.734 | 2.530 | 1.278 | 0.89 |
| LIC20148 | 0.269 | 0.353 | 2.605 | 2.093 | 2.655 | 2.925 | 2.648 | 6.119 | 2.458 | 1.70 |
| LIC20182 | 0.936 | 0.733 | 5.350 | 16.191 | 6.244 | 5.559 | 6.338 | 2.051 | 5.425 | 4.62 |
| others | 0.329 | 0.309 | 2.172 | 5.175 | 2.128 | 2.155 | 2.104 | 1.493 | 1.983 | 1.42 |

| KIDNEY |  |  |  |  |  |  |  |  |  |  |
| --- | --- | --- | --- | --- | --- | --- | --- | --- | --- | --- |
| **Animal** | **1** | **2** | **3** | **4** | **5** | **6** | **7** | **8** | **Mean** | **SD** |
| LIC10024 | 0.145 | 1.313 | 1.227 | 0.824 | 1.250 | 1.389 | 1.422 | 2.138 | 1.213 | 0.53 |
| LIC10132 | 0.331 | 0.156 | 1.745 | 0.294 | 1.471 | 1.679 | 1.719 | 5.239 | 1.579 | 1.53 |
| LIC10138 | 0.191 | 1.091 | 1.434 | 1.110 | 1.203 | 1.378 | 1.394 | 0.190 | 0.999 | 0.48 |
| LIC10191 | 0.577 | 0.189 | 2.024 | 1.859 | 1.869 | 1.906 | 2.002 | 0.873 | 1.413 | 0.69 |
| LIC10203 | 0.045 | 0.036 | 0.379 | 0.068 | 0.315 | 0.408 | 0.389 | 0.055 | 0.212 | 0.16 |
| LIC10225 | 0.275 | 0.257 | 2.539 | 0.412 | 2.137 | 2.553 | 2.492 | 0.353 | 1.377 | 1.06 |
| LIC10464 | 0.255 | 1.802 | 2.337 | 0.468 | 2.406 | 2.209 | 2.396 | 2.195 | 1.758 | 0.83 |
| LIC10641 | 5.608 | 1.959 | 3.509 | 12.068 | 4.362 | 3.842 | 2.995 | 2.340 | 4.585 | 3.03 |
| Inter10855 | 2.432 | 0.337 | 1.451 | 2.402 | 1.330 | 1.444 | 1.582 | 4.321 | 1.912 | 1.10 |
| LIC10788 | 1.729 | 8.596 | 3.802 | 4.455 | 3.590 | 3.964 | 3.667 | 4.540 | 4.293 | 1.82 |
| Inter11063 | 3.115 | 0.135 | 1.410 | 0.204 | 1.123 | 1.453 | 1.527 | 2.258 | 1.403 | 0.92 |
| LIC11081 | 6.618 | 0.204 | 2.190 | 0.432 | 1.906 | 2.175 | 2.217 | 1.382 | 2.141 | 1.85 |
| LIC11095 | 0.403 | 3.376 | 1.722 | 0.332 | 1.645 | 1.660 | 1.865 | 0.306 | 1.414 | 0.98 |
| LIC11274 | 0.182 | 6.132 | 1.681 | 0.319 | 1.369 | 1.682 | 1.614 | 1.382 | 1.795 | 1.73 |
| LIC11432 | 4.123 | 4.218 | 4.921 | 0.797 | 4.699 | 5.100 | 4.759 | 0.885 | 3.688 | 1.67 |
| LIC11563 | 0.777 | 1.907 | 2.993 | 0.620 | 4.431 | 2.771 | 3.017 | 13.174 | 3.711 | 3.76 |
| LIC11889 | 1.945 | 3.818 | 2.190 | 7.744 | 2.750 | 2.280 | 1.938 | 9.628 | 4.037 | 2.78 |
| LIC11940 | 0.782 | 0.179 | 1.437 | 0.712 | 1.156 | 1.316 | 1.445 | 0.940 | 0.996 | 0.41 |
| LIC12031 | 8.563 | 0.260 | 1.775 | 0.347 | 1.652 | 1.682 | 1.600 | 1.720 | 2.200 | 2.48 |
| LIC12218 | 3.911 | 6.233 | 3.079 | 4.485 | 2.985 | 2.852 | 3.068 | 4.065 | 3.835 | 1.07 |
| LIC12324a | 0.221 | 1.809 | 1.819 | 0.611 | 1.677 | 1.887 | 1.783 | 0.383 | 1.274 | 0.68 |
| LIC12324b | 0.092 | 0.084 | 0.828 | 0.121 | 0.706 | 0.801 | 0.942 | 1.438 | 0.627 | 0.46 |
| LIC12327a | 0.148 | 0.141 | 1.252 | 0.221 | 1.185 | 1.344 | 1.367 | 0.208 | 0.733 | 0.56 |
| LIC12327b | 0.085 | 0.581 | 0.867 | 0.140 | 0.667 | 0.679 | 0.796 | 0.609 | 0.553 | 0.27 |
| LIC12502 | 0.282 | 0.174 | 1.922 | 1.143 | 1.772 | 1.981 | 1.838 | 0.787 | 1.237 | 0.70 |
| LIC12506 | 5.281 | 2.886 | 2.445 | 6.943 | 2.525 | 2.338 | 2.437 | 2.748 | 3.450 | 1.60 |
| Inter12760 | 3.903 | 0.123 | 1.285 | 0.198 | 1.051 | 1.333 | 1.212 | 0.186 | 1.161 | 1.15 |
| LIC12627a | 0.327 | 4.525 | 3.065 | 0.523 | 2.677 | 3.117 | 3.145 | 0.585 | 2.246 | 1.46 |
| LIC12627b | 7.870 | 0.306 | 3.101 | 0.537 | 4.999 | 3.346 | 3.168 | 2.159 | 3.186 | 2.28 |
| LIC12670 | 1.107 | 0.459 | 2.811 | 4.207 | 2.735 | 2.814 | 2.780 | 6.580 | 2.937 | 1.75 |
| LIC12772 | 0.310 | 0.927 | 2.794 | 0.728 | 2.703 | 2.656 | 3.004 | 1.304 | 1.803 | 1.02 |
| LIC12773 | 0.074 | 0.082 | 0.598 | 5.925 | 0.917 | 0.652 | 0.576 | 0.114 | 1.117 | 1.84 |
| LIC13004 | 0.424 | 12.950 | 3.824 | 3.155 | 3.905 | 4.158 | 4.293 | 0.596 | 4.163 | 3.63 |
| LIC13073 | 3.151 | 0.621 | 3.218 | 4.611 | 3.453 | 3.209 | 3.305 | 2.377 | 2.993 | 1.06 |
| LIC13074 | 0.338 | 6.359 | 3.074 | 2.132 | 2.782 | 3.098 | 3.077 | 0.444 | 2.663 | 1.76 |
| Inter13512 | 1.649 | 0.220 | 2.509 | 0.529 | 1.953 | 2.361 | 2.130 | 0.353 | 1.463 | 0.89 |
| LIC13274 | 0.054 | 3.674 | 0.407 | 0.105 | 0.500 | 0.429 | 0.393 | 0.067 | 0.704 | 1.14 |
| Inter13722 | 21.252 | 7.185 | 5.128 | 11.454 | 5.854 | 4.998 | 5.340 | 10.436 | 8.956 | 5.19 |
| LIC20111 | 5.800 | 3.907 | 3.304 | 7.772 | 3.590 | 3.290 | 3.223 | 0.613 | 3.937 | 1.96 |
| Inter20138 | 0.407 | 3.311 | 1.794 | 0.320 | 1.489 | 1.744 | 1.705 | 3.367 | 1.767 | 1.06 |
| LIC20148 | 0.287 | 1.256 | 2.600 | 1.498 | 2.163 | 2.528 | 2.688 | 0.382 | 1.675 | 0.91 |
| LIC20182 | 4.561 | 4.148 | 5.389 | 6.169 | 4.818 | 5.201 | 5.331 | 5.745 | 5.170 | 0.61 |
| others | 0.371 | 2.074 | 2.118 | 1.007 | 2.232 | 2.293 | 2.359 | 0.534 | 1.624 | 0.79 |

| LIVER |  |  |  |  |  |  |  |  |  |  |
| --- | --- | --- | --- | --- | --- | --- | --- | --- | --- | --- |
| **Animal** | **1** | **2** | **3** | **4** | **5** | **6** | **7** | **8** | **Mean** | **SD** |
| LIC10024 | 2.149 | 1.464 | 1.448 | 2.814 | 0.322 | 1.212 | 1.428 | 1.428 | 1.53 | 0.67 |
| LIC10132 | 2.171 | 0.876 | 1.828 | 1.649 | 0.398 | 1.576 | 1.702 | 1.559 | 1.47 | 0.53 |
| LIC10138 | 0.684 | 0.562 | 1.571 | 1.021 | 0.287 | 1.116 | 1.258 | 0.484 | 0.87 | 0.41 |
| LIC10191 | 0.958 | 0.531 | 2.200 | 0.347 | 0.405 | 1.610 | 1.795 | 0.251 | 1.01 | 0.71 |
| LIC10203 | 0.232 | 0.036 | 0.439 | 0.340 | 0.089 | 0.337 | 0.398 | 0.007 | 0.23 | 0.16 |
| LIC10225 | 1.488 | 1.378 | 2.691 | 0.296 | 0.906 | 2.661 | 2.341 | 1.123 | 1.61 | 0.82 |
| LIC10464 | 3.079 | 1.584 | 2.265 | 5.319 | 0.488 | 2.001 | 2.561 | 4.626 | 2.74 | 1.48 |
| LIC10641 | 2.176 | 3.435 | 2.717 | 1.783 | 0.729 | 14.684 | 3.194 | 3.499 | 4.03 | 4.12 |
| Inter10855 | 1.718 | 1.867 | 1.438 | 1.648 | 0.326 | 1.266 | 1.619 | 1.926 | 1.48 | 0.48 |
| LIC10788 | 1.872 | 1.606 | 3.567 | 1.894 | 1.053 | 3.388 | 3.690 | 2.108 | 2.40 | 0.94 |
| Inter11063 | 0.632 | 0.032 | 1.495 | 0.046 | 0.305 | 1.262 | 1.160 | 0.026 | 0.62 | 0.57 |
| LIC11081 | 1.146 | 2.523 | 2.179 | 3.948 | 0.462 | 1.945 | 2.357 | 3.096 | 2.21 | 1.01 |
| LIC11095 | 0.802 | 3.712 | 1.821 | 3.439 | 0.375 | 1.535 | 1.924 | 2.116 | 1.97 | 1.08 |
| LIC11274 | 2.348 | 2.697 | 1.687 | 2.161 | 0.369 | 1.231 | 1.619 | 1.830 | 1.74 | 0.67 |
| LIC11432 | 2.008 | 2.527 | 3.879 | 2.603 | 1.081 | 5.324 | 4.686 | 2.835 | 3.12 | 1.32 |
| LIC11563 | 2.971 | 1.825 | 3.250 | 3.379 | 0.651 | 2.459 | 3.132 | 4.062 | 2.72 | 0.99 |
| LIC11889 | 3.237 | 2.553 | 2.095 | 3.288 | 0.494 | 1.680 | 2.328 | 3.966 | 2.46 | 1.01 |
| LIC11940 | 0.735 | 1.557 | 1.348 | 1.601 | 0.309 | 1.204 | 1.465 | 0.847 | 1.13 | 0.43 |
| LIC12031 | 3.565 | 2.044 | 1.741 | 1.487 | 11.851 | 1.593 | 1.615 | 2.857 | 3.34 | 3.29 |
| LIC12218 | 4.522 | 4.923 | 3.185 | 4.501 | 0.664 | 2.438 | 3.329 | 4.020 | 3.45 | 1.30 |
| LIC12324a | 1.051 | 0.047 | 2.303 | 0.069 | 0.386 | 1.732 | 1.689 | 0.034 | 0.91 | 0.85 |
| LIC12324b | 0.455 | 0.049 | 1.031 | 0.031 | 0.187 | 0.787 | 0.818 | 0.018 | 0.42 | 0.38 |
| LIC12327a | 0.869 | 0.212 | 1.556 | 0.587 | 0.277 | 1.235 | 1.320 | 0.892 | 0.87 | 0.46 |
| LIC12327b | 0.433 | 0.714 | 0.872 | 0.523 | 0.148 | 0.637 | 0.716 | 0.534 | 0.57 | 0.21 |
| LIC12502 | 1.802 | 0.681 | 2.274 | 1.953 | 1.914 | 1.799 | 1.880 | 0.783 | 1.64 | 0.54 |
| LIC12506 | 5.659 | 4.097 | 2.163 | 4.576 | 0.552 | 1.939 | 2.480 | 3.009 | 3.06 | 1.53 |
| Inter12760 | 0.662 | 0.681 | 1.542 | 0.044 | 0.265 | 1.135 | 1.278 | 0.123 | 0.72 | 0.52 |
| LIC12627a | 4.522 | 1.164 | 3.257 | 1.317 | 0.625 | 2.351 | 2.849 | 3.311 | 2.42 | 1.23 |
| LIC12627b | 1.922 | 1.767 | 2.860 | 1.796 | 0.708 | 3.128 | 3.088 | 2.390 | 2.21 | 0.77 |
| LIC12670 | 1.115 | 2.618 | 2.358 | 2.573 | 0.623 | 2.953 | 2.735 | 1.345 | 2.04 | 0.82 |
| LIC12772 | 1.364 | 5.877 | 2.817 | 4.701 | 0.573 | 2.497 | 2.926 | 3.128 | 2.99 | 1.58 |
| LIC12773 | 2.637 | 0.414 | 0.552 | 2.470 | 0.133 | 0.475 | 0.762 | 0.200 | 0.96 | 0.94 |
| LIC13004 | 3.639 | 6.767 | 3.312 | 5.513 | 3.680 | 2.917 | 4.063 | 6.795 | 4.59 | 1.45 |
| LIC13073 | 4.288 | 4.861 | 2.756 | 4.704 | 2.746 | 3.203 | 3.335 | 4.491 | 3.80 | 0.82 |
| LIC13074 | 3.066 | 1.874 | 3.643 | 0.957 | 0.635 | 2.511 | 2.816 | 1.380 | 2.11 | 1.00 |
| Inter13512 | 1.898 | 0.434 | 2.798 | 1.044 | 0.484 | 1.924 | 2.266 | 1.455 | 1.54 | 0.79 |
| LIC13274 | 0.357 | 0.894 | 0.354 | 0.467 | 0.080 | 0.356 | 0.394 | 0.987 | 0.49 | 0.28 |
| Inter13722 | 12.297 | 10.673 | 5.000 | 9.404 | 43.859 | 4.646 | 5.775 | 10.714 | 12.80 | 12.05 |
| LIC20111 | 3.277 | 4.540 | 2.959 | 2.729 | 0.679 | 2.867 | 3.157 | 3.777 | 3.00 | 1.03 |
| Inter20138 | 1.049 | 1.869 | 1.889 | 1.817 | 7.464 | 1.626 | 1.816 | 1.475 | 2.38 | 1.94 |
| LIC20148 | 3.164 | 2.024 | 2.768 | 2.095 | 0.555 | 2.355 | 2.594 | 2.127 | 2.21 | 0.72 |
| LIC20182 | 4.775 | 4.437 | 5.772 | 2.852 | 10.997 | 4.498 | 5.078 | 3.425 | 5.23 | 2.34 |
| others | 1.203 | 5.578 | 2.320 | 4.214 | 0.864 | 1.907 | 2.565 | 4.943 | 2.95 | 1.64 |
