# Supplementary material for: High-Throughput Parallel Sequencing to Measure Fitness of Leptospira interrogans Transposon Insertion Mutants during Acute Infection
Source: PLoS Negl Trop Dis. 2016 Nov 8;10(11):e0005117. doi: 10.1371/journal.pntd.0005117 (PMC5100919; doi:10.1371/journal.pntd.0005117)
Supplement: S3 Table — Median of ratios and variance per mutant. (DOCX) [file pntd.0005117.s003.docx]

**S3 Table. Output/input ratios** of each mutant frequencies in each animal in blood, kidney and liver. Median of ratios and variance per mutant.

| BLOOD |  |  |  |  |  |  |  |  |  |  |
| --- | --- | --- | --- | --- | --- | --- | --- | --- | --- | --- |
| **Animal** | **1** | **2** | **3** | **4** | **5** | **6** | **7** | **8** | **Median** | **Variance** |
| LIC10024 | 1.246 | 0.858 | 0.865 | 0.875 | 0.932 | 0.823 | 0.907 | 0.585 | 0.870 | 0.03 |
| LIC10132 | 2.719 | 0.837 | 0.814 | 0.776 | 0.971 | 0.800 | 0.929 | 0.602 | 0.826 | 0.41 |
| LIC10138 | 11.616 | 0.657 | 0.600 | 0.674 | 0.836 | 0.670 | 0.795 | 0.909 | 0.735 | 12.96 |
| LIC10191 | 0.313 | 0.604 | 0.686 | 0.688 | 0.817 | 0.756 | 0.820 | 0.505 | 0.687 | 0.03 |
| LIC10203 | 0.207 | 0.527 | 0.529 | 0.503 | 0.767 | 0.484 | 0.760 | 0.402 | 0.515 | 0.03 |
| LIC10225 | 0.679 | 1.312 | 1.255 | 1.188 | 1.193 | 1.378 | 1.175 | 18.808 | 1.224 | 34.07 |
| LIC10464 | 1.666 | 0.992 | 1.030 | 0.977 | 0.990 | 0.971 | 0.981 | 0.925 | 0.986 | 0.05 |
| LIC10641 | 1.041 | 143.921 | 1.038 | 0.994 | 1.010 | 1.026 | 0.946 | 0.871 | 1.018 | 2234.46 |
| Inter10855 | 1.061 | 1.342 | 1.430 | 242.570 | 1.409 | 1.445 | 1.292 | 1.074 | 1.375 | 6367.23 |
| LIC10788 | 22.616 | 1.465 | 1.119 | 21.039 | 1.075 | 1.126 | 1.058 | 1.436 | 1.281 | 79.85 |
| Inter11063 | 0.341 | 0.794 | 0.780 | 0.719 | 0.854 | 0.780 | 0.872 | 0.580 | 0.780 | 0.03 |
| LIC11081 | 13.910 | 1.000 | 0.939 | 0.984 | 0.973 | 0.975 | 0.963 | 0.661 | 0.974 | 18.45 |
| LIC11095 | 0.622 | 0.991 | 0.942 | 40.350 | 1.046 | 1.068 | 1.070 | 0.796 | 1.018 | 169.95 |
| LIC11274 | 1.122 | 1.341 | 1.222 | 122.122 | 1.251 | 1.435 | 1.178 | 19.746 | 1.296 | 1565.33 |
| LIC11432 | 1.360 | 2.358 | 2.498 | 2.107 | 1.596 | 2.137 | 1.668 | 13.111 | 2.122 | 13.73 |
| LIC11563 | 0.603 | 0.776 | 0.817 | 0.786 | 0.974 | 0.864 | 0.922 | 28.064 | 0.841 | 81.19 |
| LIC11889 | 2.981 | 66.690 | 1.161 | 1.121 | 1.112 | 1.200 | 1.077 | 7.858 | 1.180 | 457.42 |
| LIC11940 | 0.566 | 1.045 | 1.040 | 0.993 | 1.040 | 1.049 | 1.080 | 73.423 | 1.043 | 574.12 |
| LIC12031 | 0.618 | 0.863 | 0.908 | 0.799 | 0.934 | 0.814 | 0.883 | 0.598 | 0.839 | 0.01 |
| LIC12218 | 3.816 | 1.088 | 1.045 | 59.818 | 1.146 | 1.168 | 1.023 | 0.765 | 1.117 | 373.65 |
| LIC12324a | 0.275 | 0.581 | 0.563 | 0.566 | 0.744 | 0.594 | 0.781 | 0.455 | 0.574 | 0.02 |
| LIC12324b | 0.284 | 0.562 | 0.592 | 0.611 | 0.859 | 0.647 | 0.794 | 0.495 | 0.602 | 0.03 |
| LIC12327a | 0.363 | 0.680 | 0.711 | 0.667 | 0.813 | 0.738 | 0.837 | 0.540 | 0.695 | 0.02 |
| LIC12327b | 0.308 | 0.581 | 0.552 | 0.590 | 0.807 | 0.605 | 0.767 | 0.471 | 0.586 | 0.02 |
| LIC12502 | 31.692 | 0.999 | 1.035 | 0.947 | 1.162 | 1.093 | 1.021 | 8.211 | 1.064 | 101.50 |
| LIC12506 | 1.083 | 1.539 | 1.376 | 27.042 | 1.273 | 1.346 | 1.282 | 51.770 | 1.361 | 310.26 |
| Inter12760 | 0.336 | 0.736 | 0.693 | 0.751 | 0.850 | 0.721 | 0.871 | 0.516 | 0.728 | 0.03 |
| LIC12627a | 22.148 | 0.760 | 0.693 | 84.739 | 0.923 | 0.773 | 0.839 | 0.551 | 0.806 | 765.35 |
| LIC12627b | 3.794 | 1.639 | 1.679 | 1.515 | 1.496 | 1.632 | 1.320 | 1.088 | 1.573 | 0.62 |
| LIC12670 | 1.341 | 2.496 | 2.422 | 2.139 | 1.818 | 2.261 | 1.764 | 1.446 | 1.979 | 0.17 |
| LIC12772 | 0.763 | 1.115 | 1.145 | 13.187 | 1.125 | 1.179 | 1.084 | 8.497 | 1.135 | 19.30 |
| LIC12773 | 0.857 | 1.024 | 1.130 | 1.086 | 0.974 | 0.961 | 1.218 | 144.032 | 1.055 | 2236.50 |
| LIC13004 | 73.506 | 2.403 | 2.027 | 308.552 | 1.754 | 2.233 | 1.594 | 48.583 | 2.318 | 9839.57 |
| LIC13073 | 1.483 | 2.145 | 1.971 | 1.939 | 1.580 | 1.830 | 1.548 | 1.237 | 1.705 | 0.08 |
| LIC13074 | 12.803 | 0.620 | 0.586 | 20.286 | 0.789 | 0.648 | 0.799 | 0.467 | 0.719 | 50.87 |
| Inter13512 | 0.393 | 0.618 | 0.672 | 0.618 | 0.836 | 0.745 | 0.814 | 0.520 | 0.645 | 0.02 |
| LIC13274 | 0.958 | 1.163 | 1.281 | 1.004 | 0.962 | 1.148 | 1.021 | 0.762 | 1.012 | 0.02 |
| Inter13722 | 6.925 | 1.233 | 1.089 | 0.988 | 1.119 | 1.094 | 1.018 | 4.204 | 1.106 | 4.22 |
| LIC20111 | 0.740 | 32.496 | 0.923 | 0.918 | 1.010 | 0.888 | 1.003 | 34.315 | 0.963 | 198.16 |
| Inter20138 | 0.677 | 1.185 | 1.110 | 1.048 | 1.151 | 1.212 | 1.093 | 14.080 | 1.131 | 18.54 |
| LIC20148 | 0.593 | 0.933 | 0.970 | 18.876 | 1.033 | 1.117 | 0.997 | 20.339 | 1.015 | 65.49 |
| LIC20182 | 0.777 | 0.727 | 0.749 | 54.879 | 0.913 | 0.798 | 0.897 | 2.562 | 0.848 | 317.14 |

| KIDNEY |  |  |  |  |  |  |  |  |  |  |
| --- | --- | --- | --- | --- | --- | --- | --- | --- | --- | --- |
| **Animal** | **1** | **2** | **3** | **4** | **5** | **6** | **7** | **8** | **Median** | **Variance** |
| LIC10024 | 0.373 | 1.617 | 0.751 | 1.464 | 0.817 | 0.868 | 0.859 | 1.979 | 0.863 | 0.25 |
| LIC10132 | 0.641 | 0.145 | 0.806 | 0.394 | 0.726 | 0.792 | 0.784 | 3.662 | 0.755 | 1.06 |
| LIC10138 | 0.379 | 1.039 | 0.679 | 1.525 | 0.608 | 0.666 | 0.651 | 0.136 | 0.658 | 0.15 |
| LIC10191 | 0.824 | 0.130 | 0.690 | 1.839 | 0.680 | 0.663 | 0.673 | 0.450 | 0.677 | 0.21 |
| LIC10203 | 0.232 | 0.091 | 0.473 | 0.245 | 0.419 | 0.519 | 0.478 | 0.104 | 0.332 | 0.03 |
| LIC10225 | 0.578 | 0.260 | 1.274 | 0.599 | 1.144 | 1.307 | 1.233 | 0.268 | 0.872 | 0.18 |
| LIC10464 | 0.456 | 1.551 | 1.000 | 0.580 | 1.098 | 0.964 | 1.011 | 1.420 | 1.005 | 0.12 |
| LIC10641 | 8.865 | 1.489 | 1.325 | 13.220 | 1.758 | 1.481 | 1.116 | 1.337 | 1.485 | 18.58 |
| Inter10855 | 9.985 | 0.665 | 1.423 | 6.835 | 1.392 | 1.446 | 1.530 | 6.409 | 1.488 | 10.77 |
| LIC10788 | 2.184 | 5.218 | 1.147 | 3.899 | 1.156 | 1.220 | 1.091 | 2.071 | 1.646 | 2.05 |
| Inter11063 | 6.885 | 0.143 | 0.744 | 0.313 | 0.633 | 0.783 | 0.795 | 1.803 | 0.764 | 4.33 |
| LIC11081 | 12.558 | 0.186 | 0.993 | 0.568 | 0.922 | 1.006 | 0.992 | 0.948 | 0.970 | 15.19 |
| LIC11095 | 0.950 | 3.825 | 0.970 | 0.542 | 0.988 | 0.954 | 1.036 | 0.260 | 0.962 | 1.06 |
| LIC11274 | 0.596 | 9.685 | 1.320 | 0.727 | 1.147 | 1.347 | 1.250 | 1.640 | 1.285 | 8.07 |
| LIC11432 | 8.233 | 4.050 | 2.348 | 1.103 | 2.393 | 2.483 | 2.240 | 0.638 | 2.370 | 4.91 |
| LIC11563 | 0.893 | 1.054 | 0.822 | 0.494 | 1.299 | 0.777 | 0.818 | 5.472 | 0.858 | 2.35 |
| LIC11889 | 4.759 | 4.490 | 1.281 | 13.129 | 1.715 | 1.360 | 1.118 | 8.511 | 3.103 | 16.25 |
| LIC11940 | 2.421 | 0.266 | 1.063 | 1.529 | 0.913 | 0.994 | 1.054 | 1.052 | 1.053 | 0.33 |
| LIC12031 | 18.063 | 0.264 | 0.895 | 0.507 | 0.889 | 0.865 | 0.795 | 1.311 | 0.877 | 32.72 |
| LIC12218 | 5.920 | 4.536 | 1.114 | 4.705 | 1.152 | 1.053 | 1.095 | 2.224 | 1.688 | 3.52 |
| LIC12324a | 0.282 | 1.109 | 0.554 | 0.540 | 0.545 | 0.587 | 0.536 | 0.177 | 0.543 | 0.07 |
| LIC12324b | 0.272 | 0.119 | 0.587 | 0.249 | 0.534 | 0.579 | 0.658 | 1.541 | 0.557 | 0.17 |
| LIC12327a | 0.302 | 0.138 | 0.611 | 0.313 | 0.617 | 0.670 | 0.658 | 0.154 | 0.462 | 0.05 |
| LIC12327b | 0.264 | 0.868 | 0.644 | 0.302 | 0.528 | 0.515 | 0.583 | 0.684 | 0.555 | 0.03 |
| LIC12502 | 0.615 | 0.182 | 1.000 | 1.725 | 0.984 | 1.052 | 0.943 | 0.619 | 0.964 | 0.17 |
| LIC12506 | 13.406 | 3.522 | 1.483 | 12.213 | 1.634 | 1.447 | 1.458 | 2.520 | 2.077 | 22.40 |
| Inter12760 | 8.315 | 0.126 | 0.654 | 0.292 | 0.571 | 0.692 | 0.608 | 0.143 | 0.589 | 6.83 |
| LIC12627a | 0.337 | 2.239 | 0.754 | 0.373 | 0.703 | 0.782 | 0.763 | 0.217 | 0.728 | 0.35 |
| LIC12627b | 17.110 | 0.320 | 1.611 | 0.809 | 2.772 | 1.774 | 1.623 | 1.696 | 1.660 | 27.05 |
| LIC12670 | 3.930 | 0.783 | 2.383 | 10.345 | 2.475 | 2.435 | 2.325 | 8.437 | 2.455 | 10.04 |
| LIC12772 | 0.551 | 0.792 | 1.186 | 0.897 | 1.224 | 1.151 | 1.258 | 0.837 | 1.024 | 0.06 |
| LIC12773 | 0.524 | 0.278 | 1.006 | 28.907 | 1.645 | 1.118 | 0.956 | 0.289 | 0.981 | 86.40 |
| LIC13004 | 1.050 | 15.405 | 2.261 | 5.410 | 2.464 | 2.508 | 2.504 | 0.533 | 2.484 | 20.33 |
| LIC13073 | 8.243 | 0.781 | 2.011 | 8.358 | 2.303 | 2.047 | 2.038 | 2.246 | 2.147 | 7.87 |
| LIC13074 | 0.276 | 2.500 | 0.601 | 1.208 | 0.580 | 0.618 | 0.593 | 0.131 | 0.597 | 0.49 |
| Inter13512 | 1.897 | 0.121 | 0.690 | 0.422 | 0.573 | 0.662 | 0.578 | 0.147 | 0.575 | 0.27 |
| LIC13274 | 0.613 | 20.106 | 1.107 | 0.825 | 1.451 | 1.192 | 1.055 | 0.275 | 1.081 | 40.33 |
| Inter13722 | 18.658 | 3.032 | 1.076 | 6.969 | 1.311 | 1.070 | 1.105 | 3.310 | 2.171 | 31.91 |
| LIC20111 | 7.414 | 2.401 | 1.009 | 6.885 | 1.170 | 1.026 | 0.971 | 0.283 | 1.098 | 7.08 |
| Inter20138 | 1.084 | 4.241 | 1.143 | 0.591 | 1.012 | 1.133 | 1.071 | 3.242 | 1.108 | 1.49 |
| LIC20148 | 0.456 | 0.961 | 0.989 | 1.652 | 0.878 | 0.981 | 1.009 | 0.220 | 0.971 | 0.16 |
| LIC20182 | 2.729 | 1.193 | 0.770 | 2.558 | 0.735 | 0.759 | 0.752 | 1.242 | 0.982 | 0.60 |

| LIVER |  |  |  |  |  |  |  |  |  |  |
| --- | --- | --- | --- | --- | --- | --- | --- | --- | --- | --- |
| **Animal** | **1** | **2** | **3** | **4** | **5** | **6** | **7** | **8** | **Median** | **Variance** |
| LIC10024 | 1.496 | 1.076 | 0.906 | 1.852 | 0.892 | 0.844 | 0.817 | 0.964 | 0.935 | 0.12 |
| LIC10132 | 1.140 | 0.486 | 0.863 | 0.819 | 0.833 | 0.829 | 0.736 | 0.795 | 0.824 | 0.03 |
| LIC10138 | 0.368 | 0.319 | 0.760 | 0.520 | 0.616 | 0.601 | 0.557 | 0.253 | 0.538 | 0.03 |
| LIC10191 | 0.371 | 0.217 | 0.767 | 0.127 | 0.626 | 0.625 | 0.572 | 0.094 | 0.472 | 0.06 |
| LIC10203 | 0.328 | 0.055 | 0.559 | 0.455 | 0.501 | 0.479 | 0.464 | 0.010 | 0.459 | 0.04 |
| LIC10225 | 0.849 | 0.829 | 1.380 | 0.160 | 2.056 | 1.519 | 1.098 | 0.621 | 0.973 | 0.30 |
| LIC10464 | 1.497 | 0.813 | 0.990 | 2.445 | 0.945 | 0.974 | 1.024 | 2.183 | 1.007 | 0.34 |
| LIC10641 | 0.934 | 1.557 | 1.049 | 0.724 | 1.247 | 6.312 | 1.128 | 1.458 | 1.187 | 2.97 |
| Inter10855 | 1.916 | 2.198 | 1.442 | 1.738 | 1.447 | 1.413 | 1.485 | 2.084 | 1.611 | 0.09 |
| LIC10788 | 0.642 | 0.582 | 1.100 | 0.614 | 1.438 | 1.163 | 1.041 | 0.701 | 0.871 | 0.09 |
| Inter11063 | 0.379 | 0.020 | 0.807 | 0.026 | 0.729 | 0.758 | 0.573 | 0.015 | 0.476 | 0.11 |
| LIC11081 | 0.591 | 1.373 | 1.010 | 1.924 | 0.948 | 1.003 | 0.999 | 1.548 | 1.007 | 0.15 |
| LIC11095 | 0.514 | 2.509 | 1.048 | 2.081 | 0.956 | 0.983 | 1.013 | 1.315 | 1.031 | 0.38 |
| LIC11274 | 2.095 | 2.542 | 1.354 | 1.823 | 1.310 | 1.099 | 1.188 | 1.584 | 1.469 | 0.22 |
| LIC11432 | 1.089 | 1.447 | 1.892 | 1.335 | 2.335 | 2.891 | 2.090 | 1.492 | 1.692 | 0.31 |
| LIC11563 | 0.928 | 0.602 | 0.913 | 0.997 | 0.809 | 0.769 | 0.804 | 1.231 | 0.861 | 0.03 |
| LIC11889 | 2.151 | 1.791 | 1.252 | 2.066 | 1.307 | 1.118 | 1.272 | 2.557 | 1.549 | 0.24 |
| LIC11940 | 0.619 | 1.383 | 1.020 | 1.273 | 1.036 | 1.013 | 1.013 | 0.691 | 1.016 | 0.06 |
| LIC12031 | 2.043 | 1.236 | 0.897 | 0.806 | 27.032 | 0.914 | 0.761 | 1.588 | 1.075 | 73.28 |
| LIC12218 | 1.859 | 2.137 | 1.178 | 1.750 | 1.086 | 1.004 | 1.126 | 1.604 | 1.391 | 0.16 |
| LIC12324a | 0.364 | 0.017 | 0.717 | 0.023 | 0.532 | 0.601 | 0.481 | 0.012 | 0.423 | 0.07 |
| LIC12324b | 0.367 | 0.041 | 0.747 | 0.023 | 0.600 | 0.635 | 0.542 | 0.014 | 0.454 | 0.08 |
| LIC12327a | 0.482 | 0.124 | 0.777 | 0.308 | 0.612 | 0.686 | 0.603 | 0.480 | 0.543 | 0.04 |
| LIC12327b | 0.366 | 0.637 | 0.662 | 0.417 | 0.499 | 0.538 | 0.497 | 0.438 | 0.498 | 0.01 |
| LIC12502 | 1.066 | 0.425 | 1.210 | 1.093 | 4.508 | 1.065 | 0.914 | 0.449 | 1.066 | 1.51 |
| LIC12506 | 3.902 | 2.983 | 1.341 | 2.983 | 1.514 | 1.338 | 1.406 | 2.013 | 1.764 | 0.84 |
| Inter12760 | 0.383 | 0.416 | 0.802 | 0.024 | 0.611 | 0.657 | 0.608 | 0.069 | 0.512 | 0.07 |
| LIC12627a | 1.264 | 0.343 | 0.819 | 0.348 | 0.695 | 0.658 | 0.655 | 0.898 | 0.677 | 0.08 |
| LIC12627b | 1.135 | 1.102 | 1.519 | 1.003 | 1.666 | 1.849 | 1.500 | 1.369 | 1.435 | 0.08 |
| LIC12670 | 1.075 | 2.665 | 2.044 | 2.345 | 2.390 | 2.849 | 2.168 | 1.258 | 2.256 | 0.35 |
| LIC12772 | 0.658 | 2.995 | 1.222 | 2.145 | 1.100 | 1.206 | 1.161 | 1.465 | 1.214 | 0.47 |
| LIC12773 | 5.042 | 0.835 | 0.948 | 4.465 | 1.010 | 0.909 | 1.199 | 0.371 | 0.979 | 2.88 |
| LIC13004 | 2.445 | 4.802 | 2.001 | 3.503 | 9.847 | 1.963 | 2.246 | 4.431 | 2.974 | 6.10 |
| LIC13073 | 3.046 | 3.646 | 1.761 | 3.160 | 7.767 | 2.278 | 1.949 | 3.096 | 3.071 | 3.18 |
| LIC13074 | 0.681 | 0.439 | 0.728 | 0.201 | 0.562 | 0.558 | 0.514 | 0.297 | 0.536 | 0.03 |
| Inter13512 | 0.593 | 0.143 | 0.786 | 0.308 | 0.602 | 0.602 | 0.582 | 0.441 | 0.588 | 0.04 |
| LIC13274 | 1.104 | 2.917 | 0.983 | 1.365 | 0.990 | 1.102 | 1.001 | 2.960 | 1.103 | 0.65 |
| Inter13722 | 2.932 | 2.687 | 1.072 | 2.120 | 41.634 | 1.109 | 1.132 | 2.479 | 2.300 | 172.89 |
| LIC20111 | 1.138 | 1.664 | 0.924 | 0.896 | 0.939 | 0.997 | 0.902 | 1.272 | 0.968 | 0.06 |
| Inter20138 | 0.759 | 1.429 | 1.229 | 1.244 | 21.506 | 1.178 | 1.081 | 1.036 | 1.204 | 45.42 |
| LIC20148 | 1.368 | 0.924 | 1.076 | 0.856 | 0.955 | 1.019 | 0.922 | 0.893 | 0.939 | 0.02 |
| LIC20182 | 0.776 | 0.761 | 0.843 | 0.438 | 7.114 | 0.732 | 0.679 | 0.540 | 0.746 | 4.54 |
