# Supplementary material for: High-Throughput Parallel Sequencing to Measure Fitness of Leptospira interrogans Transposon Insertion Mutants during Acute Infection
Source: PLoS Negl Trop Dis. 2016 Nov 8;10(11):e0005117. doi: 10.1371/journal.pntd.0005117 (PMC5100919; doi:10.1371/journal.pntd.0005117)
Supplement: S4 Table — (DOCX) [file pntd.0005117.s004.docx]

**S4 Table. Survival experiment with selected mutants.**

| **Strain** | **Number of sacrificed animals/ number of infected animals (days of endpoint)** | **% of survivors** | **P value  compared to WT** |
| --- | --- | --- | --- |
| **WT** | 6/6 (5,5,5,5,5,8) | 0 | N/A |
| ***loa22^-^*** | 3/6 (6,8,9) | 50 | 0.0046 |
| ***flaA1^-^*** | 5/6 (5,5,5,6,7) | 17 | 0.4364 |
| ***ligB^-^*** | 6/6 (5,5,5,5,5,6) | 0 | 0.5514 |
| ***lic20111^-^*** | 6/6 (6,6,6,6,7,8) | 0 | 0.1557 |
| ***lic12327a^-^*** | 2/6 (7,7) | 67 | 0.0051 |
| ***lic12327b^-^*** | 1/6 (7) | 83 | 0.0019 |
