# Supplementary figures and images for: High-Throughput Parallel Sequencing to Measure Fitness of Leptospira interrogans Transposon Insertion Mutants during Acute Infection
Source: PLoS Negl Trop Dis. 2016 Nov 8;10(11):e0005117. doi: 10.1371/journal.pntd.0005117 (PMC5100919; doi:10.1371/journal.pntd.0005117)

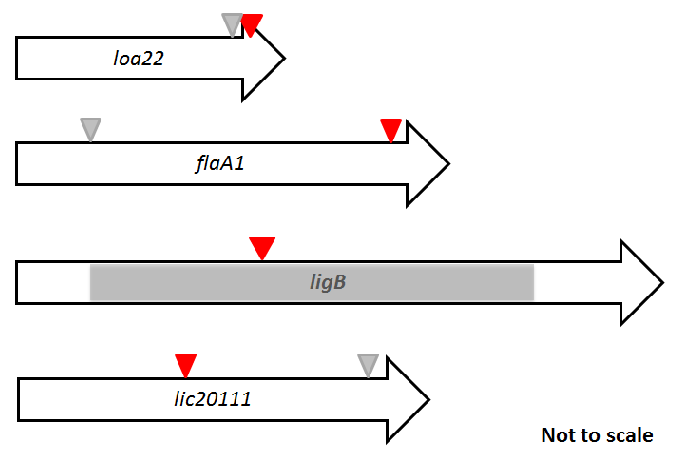

Supplement: S1 Fig — The red arrow represents the insertion site in this study and the grey one in previously described studies: Ristow et al. [11], Lambert et al. [48] and Esghi et al. [12], for loa22::Tn, flaA1::Tn and lic20111::Tn respectively. The gray zone in ligB gene shows the part of the gene that was removed in Croda et al, study [13]. (TIFF) [file pntd.0005117.s005.tiff]

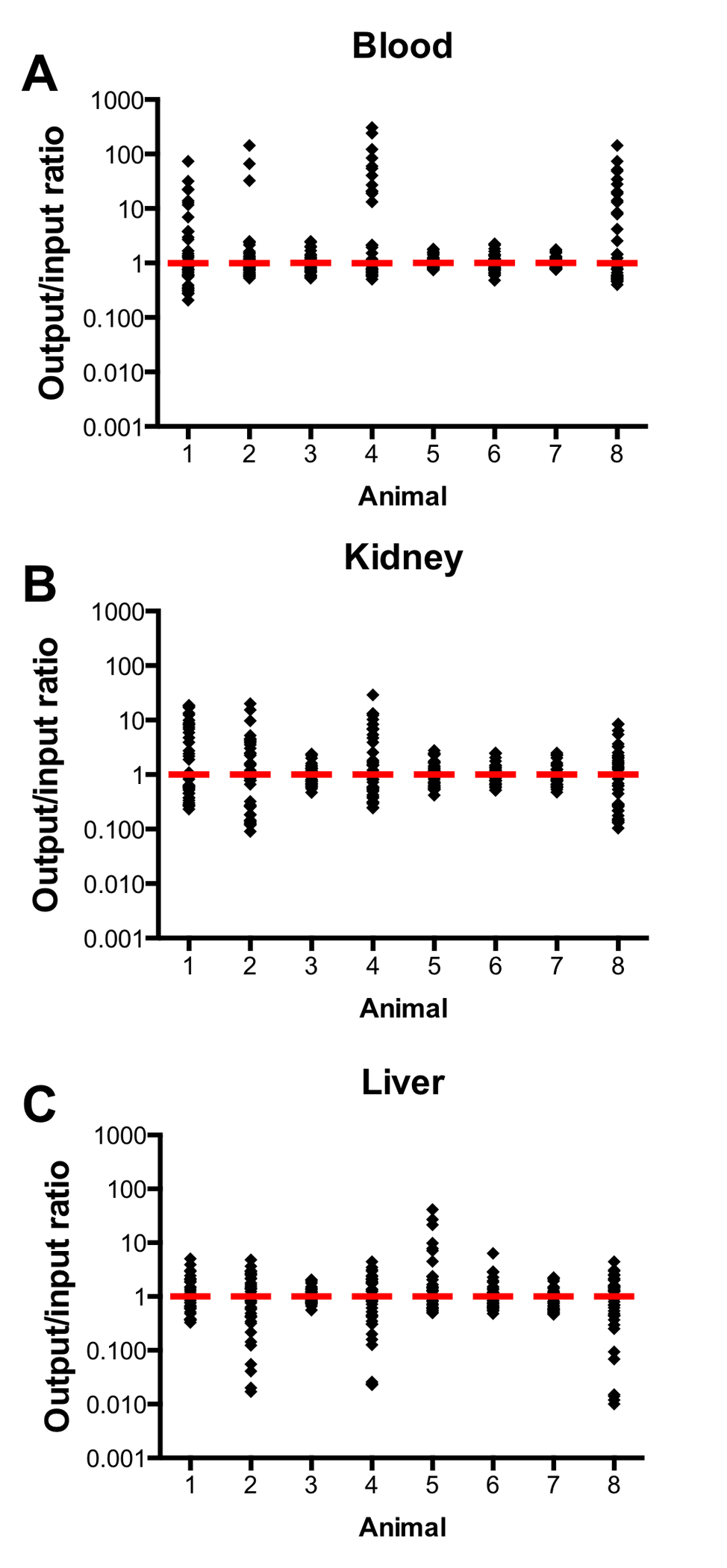

Supplement: S2 Fig — The output/input ratio of each mutant was determined for each animal in (A) blood, (B) kidney and (C) liver. The ratio of each mutant is represented by a black diamonds and the median of these ratios by a red line. (TIFF) [file pntd.0005117.s006.tiff]
